# Supplementary material for: Digital contact tracing and network theory to stop the spread of COVID-19 using big-data on human mobility geolocalization
Source: PLoS Comput Biol. 2022 Apr 11;18(4):e1009865. doi: 10.1371/journal.pcbi.1009865 (PMC9053778; doi:10.1371/journal.pcbi.1009865)
Supplement: S1 Appendix — Detailed description of of the data acquisition and treatment (Section 1), of the theoretical tools mentioned in the main text (Sections 2, 3, 4, 5 and 6), and discussion on the extent to which the present results would hold under implementation in terms of robustness to data quality and coverage, sampling bias on demographics such as coverage of location, socio-economic status, age and gender and privacy (Section 7). (PDF) [file pcbi.1009865.s001.pdf]

Supplementary Materials for

Digital contact tracing and network theory to stop the spread of COVID-19 using big-data on human mobility geolocalization

Matteo Serafino, Higor S. Monteiro, Shaojun Luo,
Saulo D. S. Reis, Carles Igual, Antonio S. Lima Neto,
Matías Travizano, José S. Andrade Jr, Hernán A. Makse*

*Correspondence to: hmakse@ccny.cuny.edu

This PDF file includes

Materials and Methods

Figures S1–S17

Table S1

Materials and Methods

S1 Datasets **4**

S2 Digital contact tracing model **6**

S3 Model calibration **7**

S4 Percolation, giant connected component and centralities **8**

S5 K-cores and k-shell decomposition of the network **10**

S6 Degree Distribution of the contact Networks **12**

S7 Implementation, sampling bias, robustness and incompleteness of the dataset **13**

S7.1 Implementation 13

S7.2 Sampling bias 16

S7.3 Correlation between a unique mobile ID and a unique person 18

S7.4 Persistence of centrality 18

S7.5 Uncertainty in the first day of symptoms 19

S7.6 Temporal sampling 19

S7.7 Temporal features 20

S7.8 Unmatched cases 20

S7.9 Sparseness of the dataset 21

S7.10Time-evolving weak ties 22

S7.11Infected cases 22

S7.12Contact tracing methods 22

S7.13K-core infection 23

S7.14Quarantines 23
S7.15Model of infection 23
S7.16Dynamics of weak links 24
S7.17Uncertainties in infected population 25
S7.18Level of uncertainty required for implementation 25
S7.19Global measures and scalability 26
S7.20Asymptomatic cases 26
S7.21Tenuous contacts 27
S7.22Quantification of uncertainty in data 27
S7.23Privacy considerations 28

S1 Datasets

The GPS dataset from 'Grandata-United Nations Development Programme partnership to combat COVID-19 with data' `https://covid.grandata.com` [1] provides the geolocation data of users of a compilation of hundreds of mobile apps across Latin America. Each datapoint registers the geohash location of a mobile ID with a 12 digits precision (cm resolution, although we use meter resolution due to noise in the determination of the exact GPS location). The mobile ID is MD5 hashed data associated with a hash of the MADID (Mobile Advertising ID).

	Ceará		
Month 2020	Unique users	Daily users	GPS Datapoints
March	111,168	43,231	153,282,989
April	145,148	49,913	161,304,623
May	158,405	63,489	221,381,875
Total	257,606	52,211	535,969,487

S1 Table GPS datasets of Ceará from [1].

See [1]: The full GPS dataset covers 10 countries across Latin America: Argentina, Brazil, Chile, Colombia, Ecuador, Guatemala, Mexico, Paraguay, Peru and Uruguay. Average number of users in the dataset per day: Uruguay: tens of thousands of unique users per day. Argentina: hundreds of thousands of unique users per day. Mexico: Millions of unique users per day. Brazil: tens of millions of unique users per day. Over the month of March, lockdowns have been imposed on each country. Pre-quarantine dates in Latin America range: March: 1st through 19th, 2020. Quarantine in Latin America dates range: March 20th till today, as of June 9.

We measure the average root mean square displacement (MSD) of the users in each country per day. Averaging over all the countries in Latin America we measure a -53.4% reduction in the MSD from a reference date before lockdown on March 6, 2020 to May 1, 2020, see [1].

In the state of Ceará, we find a reduction in mobility from MSD=988m pre-quarantine to

MSD= 430m, giving a reduction of 56.5%. The GCC of transmission contacts defined over one week window in the state of Ceará is reduced from a size of 9402 users pre-lockdown to an average size of 983 users per week during the lockdown, giving a reduction of the GCC to -10.4% from its original pre-quarantine size.

Patient's datasets are provided by the Department of Epidemiological Surveillance of the Fortaleza Health Secretariat in the city of Fortaleza, Ceará, Brazil. The research was approved by IRB at CCNY and UFC. Patient data was used with the approval and consent from the Epidemiological Surveillance Department, Fortaleza Health Secretariat at the Prefeitura de Fortaleza, Ceará, Brazil.

We cross the information from the Health Department dataset with the GPS dataset detecting the mobile ID's that are related to any confirmed case from the Health Department data using their geolocalized address. We set the night time period from 10 PM to 5 AM as a time window with high probability of being at the location. Afterwards, we identify the mobile ID of the user that spent more than 15% of the total time in the location in those time-spatial areas during March-May 2020. We assign that mobile ID to the patient.

In the Fortaleza dataset there were 8,323 cumulative cases from March 1 to May 7, 2020, out of which 5,814 contain geolocation and date of first symptoms, and 1,440 cases were matched to the GPS dataset. Lockdown started on March 19, 2020. On the month of March, 465 cases are matched which are used to calibrate the model. The number of nodes in the transmission tree of Fig 1C is 90, the number of connections in the tree is 60, and the number of secondary infections in the tree is 52. Our results can be applied to other areas as well. In preliminary studies we find that they are reproducible in other studied regions such as the state of Puebla, México. Section S7 addresses issues of sampling bias and coverage of the data relevant for implementation.

S2 Digital contact tracing model

For each time stamp starting at t_0 we draw the time-spatial area shown in Fig 1B and collect all the geolocalized data points inside the area. Afterwards we compute the average position for each user and the interval of time they have been within the contact area: $\Delta t^x = t_l^x - t_f^x$, where t_f^x and t_l^x are the first and last data points a user x (target (t) or source (s)) was within the circle.

The first component of the probability of infection is the space component [2]:

$$p_d[n] = 1 - \frac{d[n]}{2r}, \tag{1}$$

with $d[n] = |\langle s\rangle - \langle t\rangle|$, where $\langle x\rangle$ in $\{s, t\}$ refers to the average position of x inside the time-space area. That is, it is the distance between the source and target average positions of the data points within the contact area.

The time component is proportional to the overlapped amount of time that source and target spent within the contact area:

$$p_t[n] = \frac{\tau(\Delta t^s, \Delta t^t)[n]}{T}, \tag{2}$$

and

$$\tau(\Delta t^s, \Delta t^t)[n] = \begin{cases} 0, & \text{if } (\max(t_f^t, t_f^s) > \min(t_l^t, t_l^s)) \\ \max(t_f^t, t_f^s) - \min(t_l^t, t_l^s), & \text{otherwise.} \end{cases} \tag{3}$$

Here, $\tau(\Delta t^s, \Delta t_t)[n]$ is the overlapped time in the time stamp n. Note that each user needs two data points within the contact area to define its Δt^x and $\tau[n]$, otherwise $\tau[n] = 0$. When the source is the one that does not fulfill this requirement we omit that contact area.

Using both components, we define the single contact probability:

$$p_i[n] = p_d[n] \cdot p_t[n] \tag{4}$$

for each time stamp n. Using the recursive Eq. (1) we compute a unique value for each contact accumulating recursively the values of all single contacts.

S3 Model calibration

We calibrate the model to fit the basic reproduction number $R_0 = 2.78$ obtained by fitting the cumulative number of cases with the SEIR model adjusting the model hyperparameters: r, T and p_c for labeling a contact as infectious. For this calibration we trace contacts among the COVID-19 patients. We use a window of 4 days before and 7 days after the onset of symptoms which is the extended infectious period from Fig 1A. The detected contacts are labeled as infectious if the target starts with symptoms within a maximum of 14 days from the exposure. Only the first contact that fulfills the requirements is considered infectious. After running the calibration process we find the hyperparameters of the contact model that fit the R_0 estimated value.

The calibration period runs over the month of March, 2020 in the state of Ceará. In March 2020, there were 1392 infected cases reported in the state dataset localized in the city of Fortaleza. Out of these cases, we cross-checked 465 infected users in the GPS dataset. We then trace the contacts of each 465 infected users over the infectiousness period of -4d/+7d from date of first symptoms against the remaining 464 infected users. We run the calibration over a set of hyperparameters to search for the best set that most closely approximates the basic reproduction number $R_0 = 2.78$. The closest fit is obtained for: T= 30 min, r =8 m, and $p_c = 0.9$.

Using these hyperparameters, we find that 90 unique infected users participated in contact events with other infected users. That is, 90 users have either a non-zero in-degree or out-degree or both. The remaining 375 users had neither in- nor out-degree detected in the GPS database. The 90 users are plotted in the transmission tree in Fig 2B. The tree contains 60 contact events. That is, the total number of out-links (and in-links) in the tree is 60. Out of this

60 contacts, some of them coincide to the same target (see Fig 2B). Since a target can only be infected once, we do not count these duplicates to find 52 unique contacts. This results in an average out-degree for the set of located infected users of $\langle k_{\text{out}} \rangle = 52/465 = 0.112$. Since the calibration is run with a GPS dataset sample of 111,168 users out of the total population in the city of Fortaleza of 2,643,247, then we scale the average out-degree by a factor of 23.77 and obtain a calibrated $R_0^{\text{cal}} = 2.66$ which is in accordance with the estimated 2.78 value. Thus, the hyperparameters are set to $T = 30$ min, $r = 8$ m and $p_c = 0.9$.

The distribution of probabilities $P_i[n]$ from Eq. (1) shown in S1A Fig appears to be extremely polarized leaving the p_c with a wide range of optimal values for an appropriate fitting. We use p_c = 0.9 which fits our results and at the same time is large enough to filter irrelevant noisy contacts (the results do not depend largely on p_c). As shown in S1B Fig, $T = 30$ min is also the value at which the average of the contact probability $\langle P_i[n] \rangle_T$ starts to decrease with T. Therefore, $T = 30$ min is the smallest value of a well-behaved T, since we expect that the contact probability should decrease with T. Eight meters is also consistent with typical precision of geolocation given by noise level.

S4 Percolation, giant connected component and centralities

In network theory [3], the percolation problem studies the dismantling of networks under removal of nodes or links (bond percolation), as well as associated problems of disease transmission [2, 4], robustness and resilience of networks [5, 6]. The GCC is the largest connected subgraph of a graph, i.e., in the GCC there is always a pathway to reach a node from any other node. Formally, the network dismantling occurs at a critical percolation transition threshold q_c of the fraction of removed nodes q. The GCC occupies a fraction larger than zero for $q < q_c$ and vanishes otherwise. The vanishing of GCC at q_c marks the percolation transition between two phases, namely, a connected phase and a disconnected one.

One can remove nodes or links at random [3, 4] or by following optimized strategies to break the GCC with the minimal number of removals. We study different strategies to destroy the GCC with minimal removals based on removing nodes in the network by ranking them according to different centralities. All strategies are adaptive, meaning that the ranking is recalculated after every removal. We use:

(1) Degree strategy: We rank the nodes by their degree (number of contacts) from top (hubs) to low degree [5, 6] and then remove nodes starting from the hubs adaptively. We note that the process of removal of nodes from the network is a numerical trick commonly utilized to find the minimal number of hubs to dismantle the GCC. It is known in the literature that by removing the hubs adaptively, one is able to find a set of hubs that dismantle the network faster than by removing the hubs without adapting the network, see [5, 7, 8] for details. This is a purely algorithmic procedure to find the best set and the effect on the size of the GCC is the same as if one removes the nodes adaptively or at once. Thus, during the implementation of a quarantine in a real setting, the hubs obtained from the algorithm should be quarantine at once as soon as the hubs can be contacted by the contact tracer app.

(2) K-core strategy: We rank the nodes by their occupancy in the k-shells of the network. The highest rank corresponds to the inner k-shell, that is the maximal k-core at $k_{\rm core}^{\rm max}$. Since each k-shell is formed by many nodes, we then rank the nodes inside a given k-shell by their degree. We then remove each k-shell in turn, always recalculating the ranking after every removal [7].

(3) Collective Influence (CI) strategy: we calculate the CI of each node according to Ref. [8] with $\ell = 3$ and remove the nodes with CI adaptive.

(4) Betweenness Centrality (BC) strategy: We rank the nodes by their BC and remove them one by one from highest to lowest, adaptively [9, 10, 11]. We note that removing the nodes in an adaptive strategy is just a numerical trick to find a better set of spreaders. But the effect on the network is the same whether we remove the identified top spreaders one by one or all at

once. Thus, in the implementation in a real quarantine, there is no need to contact the spreaders to quarantine one by one, but they should be notified all together that they should quarantine. Furthermore, after notification, we do not remove them from the analysis, but we keep tracking them in case that they could infect new people in future networks. The betweenness centrality of a node is proportional to the number of shortest paths that pass through the node [9, 10, 11]. It is calculated by considering all the pair of nodes in the network and calculating the shortest path between each pair. This methods was found in previous simulations to be a good predictor of a node's epidemic influence in a contact network [12]. For larger datasets used in this study, approximate fast algorithms can be used to calculated BC. See Ref. [13].

(5) We also use other strategies, like eigenvector-based centralities and combinations of other centralities to characterize the node importance [14].

We test the most efficient strategy to dismantle the transmission network. We plot the normalized size of the GCC, $G(q)$, that is, the number of nodes in the GCC after removal of a fraction of q nodes divided by the size of the GCC at $q = 0$. As nodes are removed from the network, we search for the strategy that provides the minimal removal with the maximal damage to the GCC. The best strategy over all the networks studied across all of the above ranking is the high BC strategy.

S5 K-cores and k-shell decomposition of the network

The k-core of a graph is the maximal subgraph made of nodes with degree k or more. A k-shell of a graph is composed by all the nodes that belong to the k-core but not to the (k+1)-core. See S2 and S3 Figs for definitions in a network with 3-shells, i.e., with a maximal 3-core ($k_{\rm core}^{\rm max} = 3$), and S4 Fig for an example in a real network.

The k-shell decomposition assigns each node to a k-shell in the network. The k-cores are nested structures and k-shells are disjointed; e.g., the 2-core contains the 3-core and so on, and

the 2-core is formed by the 3-core plus the 2-shell. By definition, the GCC is the largest 1-core. The maximal k-core is the inner subgraph of the network and it is indexed by $k_{\rm core}^{\rm max}$ index. The low k-shell are the peripheric shells.

In practice, the k-core of a network is obtained by iteratively removing all nodes with degree smaller than k. One starts the removal process by removing all nodes of degree one (see S3 Fig). After the first removal, nodes that initially had degree larger than one may end up with degree equal to one. Then, one repeats the process until no remaining node in the network has degree equal to one, or equivalently, every node in the network has degree at least equal to 2. This set of nodes with $k = 2$, or higher, is the 2-core. The other k-cores are obtained in analogous manner.

It is important to note that a k-core can be composed of disconnected clusters or components. For instance, the example 3-core in S3C Fig contains 3 components. These three components are disconnected in the 3-core, but they are integrated in the network by nodes belonging to the 2-shell as shown in S3A Fig. This is an important property of the k-cores found in the transmission networks during the lockdowns. This property is seen in the networks studied in the main text. For instance, the network of Ceará from March 27 plotted in Fig 3B has a rich k-core structure shown in S4 Fig where, for instance, the 6-core is composed of 5 disconnected components. This is an important property for an strategy based on betweenness centrality. Typically, we find that these components are joined together by nodes in lower k-shells, which are identified by their high betweenness centrality. Then, the k-cores components can be relatively easily dismantled by a few removals outside the k-cores.

Notice that the maximal k-cores are composed of nodes with high degree that connect with other nodes of high degree, which in turn also connect with others of high degree, and so forth. This implies that the k-cores do not have dangling ends made of nodes with degree smaller than k. That is, the k-cores are close, in a sense. The k-shell decomposition then cleans the network

of those low degree dangling ends in a systematic way and reveals the core of the network, which is the most important part for spreading. See an interpretation of k-core in terms of ecosystem stability at Nature Phys. 15, 95-102 (2019).

Notice also that a hub can be in the maximal k-core or in an outer k-shell according to how the hub is connected. For instance, the red hub in S3A Fig is in the 3-core because it is also connected to other hubs with 3 or more connections. However, the orange hub is in the 1-shell in S3A Fig because it is connected with nodes with low degree.

Monitoring the k-cores of the networks as a function of time we find that before the lockdown the maximal k-core index is in average around $k_{\rm core}^{\rm max} \approx 12$ and then drops to half of this value with a maximal 6-core in average during the lockdown ($k_{\rm core}^{\rm max} \approx 6$). S6 Fig shows this drop in the maximum k-core index from $k_{\rm core}^{\rm max} \approx 12$ to $k_{\rm core}^{\rm max} \approx 6$, in average. Since $k_{\rm core}^{\rm max}$ changes with time, to study the occupancies of the k-cores and k-shells across the quarantine transition, we define the $\epsilon-$core as the k-core with k such that $k = \lceil \epsilon \, k_{\rm core}^{\rm max} \rceil$. The complement of the ϵ-core is the ϵ-shell defined as the union of the remaining k-shells with k such that $k = 1, 2, ..., \lceil \epsilon \, k_{\rm core}^{\rm max} \rceil - 1$. Thus, the union of the ϵ-core and the ϵ-shell constitute all the network. In the paper we consider $\epsilon = 0.5$ that divides the k-shells in two.

S6 Degree Distribution of the contact Networks

We analyzed the degree distribution of the contact networks before and after the quarantine. We found a relatively broad distribution as it is shown in S1 Fig, both before and after the quarantine. In addition, we find that after the quarantine the distribution is less broad than before the quarantine, displaying a smaller maximum degree. This finding is consistent with the fact that the lockdown restricts the mobility with a subsequent decrease in the maximum degree of the nodes.

S7 Implementation, sampling bias, robustness and incompleteness of the dataset

This section addresses concerns regarding the extent to which the present results would hold under implementation in terms of robustness to data quality and coverage, sampling bias on demographics such as coverage of location, socio-economic status, age and gender and privacy. Some of these issues are discussed elsewhere [12, 15].

S7.1 Implementation

Regarding the implementation of the algorithms in a real setting, we note that our algorithms are implemented following the guidelines of the WHO for developing contact tracing programs using traditional or digital technologies. The document *"Managing and monitoring contacts daily"* published in *"Contact tracing in the context of COVID-19", World Health Organization, Interim Guidance, May 2020* specifically recommends a daily monitoring of infected people, daily tracing of their contacts and daily application of quarantines based on the identified contacts.

Thus, our protocol could be implemented by local authorities by the use of a mobile app, which would (i) first identify the infected people day by day, (ii) then build the contact network back in time starting from those infections, (iii) compute if a user is a potential spreader, and (iv) if so, notify the user to quarantine, after which the user keeps being monitored.

The intervention strategy can be implemented as follows:

1. Monitor daily the number of infected people in the database. Every day the database is updated with the new cases including the information on the patient's first date of symptoms.

2. Starting from the day of first symptoms of each patient, monitor back in time and in future

days the number of contacts using digital contact tracing. From the identified first layer of contacts, obtain the chain of transmission by constructing the possible contact network upto a given number of layers. This is the transmission network at day t. This network takes into account possible pathways of the spreading.

3. After identifying all members of the transmission network, notify them of possible exposures. Additionally, perform network techniques to identify the people to dismantle the GCC and further inform them to quarantine, isolate, test or vaccinate. Optimization is applied numerically at this stage, by following the analysis in Fig 4B over different dismantling strategies. Send a message via mobile app to isolate to everyone in the transmission chain, in particular to those in the first layer of contact. Send also a message via mobile app to the obtained list of top spreaders to further suggest to quarantine. The quarantined people should not be removed from the analysis in the subsequent networks at time $t + 1$ since the quarantined person could still create new contacts in the future, if the quarantine is not followed strictly. The quarantined people are not removed from the analysis and their trajectories are being monitored in future times to detect future contacts.

4. At day $t + 1$ repeat the same procedure from Point 1-3. Add to the database the new number of reported cases at day $t + 1$ and build the new contact network explained in Point 2.

5. Repeat the above procedure daily for maximum effectivity.

Network reconstruction.– The infected people have been informed to quarantine since they are infected, tested and with symptoms and have been informed by the medical authorities to isolate. Furthermore, since our data have been acquired under the most strict mass quarantine

imposed by the authorities, everyone in the city of Fortaleza has been already informed to quarantine, including the contacts of the infected people. Indeed, the GCC is reduced to 10% and the mobility to 40%. Despite all these isolation efforts the pandemic persists. Our comprehensive contact tracing algorithm identifies further k-core superspreader events which kept the spreading.

The crucial feature of Covid-19 is that the peak of infectiousness occurs before the onset of symptoms, as shown in Fig 1A (see He, X. et al. [16]). Thus, after a person reports symptoms or tests positive, contact tracing goes back in time to capture past contacts in the past GCC.

For instance, by capturing the contacts today and in the past, since there is a delay of up to 12 days of incubation and between 3 to 10 days to infectiousness (latency, see Fig 1A), then we are effectively stopping the transmission chain 'in the future'. This situation is further clarified in S7 Fig. The infected person produces an exposure at first contact today or in the past, labeled as 'Potential Target 1st layer' in the figure. This first layer contact then enters into the infectious period anywhere between 3 to 10 days later, and produces a second layer contact as shown in the figure, thus 'propagating' the contact network to the future. These contacts 'in the future of today' are considered in the network only when we move forward in time the window of observation. That is, they are considered when the 'future contacts' are already in the past of the observation time.

When the infected person reports the symptoms, even if she/he enters into strict isolation, he/she already infected most of her/his contacts. The same happens for the contacts of the contacts and so on. The situation is worst due to the asymptomatic cases (which are captured by our model). People who get sick in the future have been infected in the past and one needs to reconstruct the network of contacts using these information of past contacts which then propagates into the future as time moves on.

The optimality in our approach is obtained by comparing the different strategies to dismantle

the GCC as in Fig 4B. While other more optimal interventions could be devised by taking into account the fact that intervening on nodes active initially is better than intervening on nodes more active at a much later point, strategies following the contacts and applying interventions as the contacts are created may be difficult to implement in real time. Therefore, our approach is not the optimal quarantine, but represents an optimized strategy to dismantle the GCC as compared with the rest of the network centralities studied. In fact, applying interventions one by one, may lead to even more efficient quarantines than those found here.

S7.2 Sampling bias

Uniformity of data coverage of the dataset across socio-economical classes, age groups, and geographical regions may affect our results due to the relatively low coverage of the GPS data. This problem is important given that the sample of the population is relatively small compared to the underlying population: 111,168 users out of the total population in the city of Fortaleza of 2,643,247 at 4% of the population and the biases could be substantial. Further, this problem is important for COVID-19 which itself has non-uniform incidence across these factors. Thus, we have investigated the sampling bias of our GPS population `https://en.wikipedia.org/wiki/Sampling_bias`.

We investigate the most likely bias dominated by geographical coverage, socio-economic status (wealthier over-represented) and age and gender of users (younger over-represented) since one would expect the majority of symptomatic cases are in the lower end of the socio-economic spectrum, and older age groups. We quantify these biases in the GPS dataset from apps by assigning each mobile app user to a geolocalized residential area defined as the place where the user spends most of the time at night between the hours of 10 PM and 5 AM in the period of study. Using these data we study the distribution of geographical localization of the app users. We consider the 120 neighborhoods (quarter or 'bairro' in Portuguese) defined by the

administrative boundaries in Fortaleza. Since the neighborhoods are extensive, the geolocalization of the users is non-identifiable. The population of each neighbourhood is provided by the Instituto Brasileiro de Geografia e Estatistica (IBGE or Census Bureau). By using the geolocation of each GPS user we calculate the fraction of app users in each neighborhood and then compare with the real fraction of the population of each neighborhood obtained from IBGE. The distribution of the populations obtained from GPS data and the real population distribution from IBGE are shown in S8 Fig. We perform a two-sample Kolmogorov-Smirnov (KS) test and find p-value =0.388, KS distance = 0.117, indicating that we cannot reject the hypothesis that the GPS data and the real data come from the same distribution. Therefore, we conclude that the GPS data has an acceptable geographical coverage of the real population indicating no sampling bias in geolocation under a statistical test.

Furthermore each neighborhood has a distinct Human Development Index ($0 \leq$HDI≤ 1) provided as well by IBGE. By using this metric of socio-economic status, we study the possibility of socio-economic bias in the population sample. We now cluster the neighborhoods by their HDI and plot the PDF and CDF of the population of neighborhoods (measured as the fraction to the total population) with a given HDI obtained from the GPS data sample and from the real population from IBGE. Results are shown in S9 Fig. We performed a two-sample Kolmogorov-Smirnov test and find that the sample distribution of HDI obtained from the GPS data and the real population distribution of HDI socio-economic status pass the KS test, p-value $= 0.699$, KS distance = 0.250. Thus, we cannot reject the hypothesis that the GPS and real data come from the same distribution indicating lack of sampling bias

The two final tests of sampling bias are done on the age distribution and the gender distribution. We do not have direct access to the age and gender of the GPS users. However, an indirect test of sampling bias can be used using the patient data. We compare the distribution of age and gender in the full patient dataset with the distribution of age and gender of those patients

that are localized in the GPS dataset. The hypothesis is that if the GPS dataset is biased by age or gender (for instance, if the app users over-represent younger people) then the distribution of localized patients in the GPS dataset should reflect this bias respect to the distribution of age and gender of the whole patient population. S10 Fig shows the respective PDF and CDF. We find that we cannot reject the hypothesis that the CDF of age from GPS data and the patient data come from the same distribution with p-value = 0.785 and KS distance = 0.039, indicating good coverage of age distribution (S10A and S10B Figs). The distributions of gender shown in S11A and S11B Figs indicate also the lack of bias in the gender distributions.

We conclude that the GPS sample does not have significant bias under statistical testing in the considered demographic variables.

S7.3 Correlation between a unique mobile ID and a unique person

To test this correlation we investigated whether the geolocalized patients in the GPS datasets have (1) visited any hospital (near 200 m) or (2) have visited any pharmacy on the date of test results. We find that out of the 1,440 patients identified in the GPS dataset 1,224 (85%) have been identified with the second test.

S7.4 Persistence of centrality

It is of interest to discuss the persistence of centrality to see how stable is that set of central people. For instance, are the same people in the maximal k-core for all time or are we using a static network metric over the aggregated network? We have calculated the persistence of people in the different k-cores and find that they persist in the networks as a function of time. Results are shown in S12 Fig.

S7.5 Uncertainty in the first day of symptoms

The day of first symptoms is the date reported retrospectively by the patient of appearance of symptoms at the first consultation at a healthcare facility, thus, there is some uncertainty in its determination related to the patient's report of this date. This uncertainty could affect the results of the contact tracing. For proper functioning of the algorithms, data should be fed into the algorithm of contact tracing in real time, while for most of the cases the first date of symptoms is reported retroactively. In a mobile app implementation of contact tracing, the user should be given the capability to report the symptoms in real time as soon as they develop, via the app, thus diminishing uncertainties in the proper definition of the window of observation to detect contacts.

To verify the reliability of the protocol, we conduct a numerical study with the random removal of infected nodes to mimic false positives on diagnosis. S13 Fig shows the relative error in the determination of the minimal number of people to quarantine as a function of false positives in the report of infected people. A false positive is an individual who reported to have symptoms but was not infected with Covid-19.

S7.6 Temporal sampling

The distribution of temporal sampling of GPS ping datapoints per user in our GPS dataset displays four peaks: around zero, at 5 minutes, 10 minutes and 20 minutes, see S14 Fig. This is consistent with other apps using, e.g., the Google-Apple framework. Typically, this is a trade-off between accuracy and battery life. In our data, pinning distributions are uniform across day and night. We have investigated the robustness of our contact tracer to different ping intervals. The effect is particularly important since betweenness centrality and k-core are both macroscopic properties, meaning a small change in the network can create large changes everywhere in the network.

S1B Fig shows that the a minimal time interval that captures the correct behaviour in the probability to find a contact is around 30 minutes. This is seen in S1B Fig where the correct decreasing behaviour of $< P_i[n] >_T$ with T appears after $T > 30$ minutes. Notice that this probability is supposed to decrease with increasing T. Considering T=30 minutes as a minimal time interval to find a contact, we would need at least two pings inside 30 minutes to properly define an interval of contacts. Therefore, we do not recommend to use longer ping intervals than 15 minutes, in order to have enough statistics to capture contact points.

S7.7 Temporal features

Bias in the temporal features of the model. The periods of infectionness and exposure are relaxed conservatively as explained in the text. We studied the robustness under the definition of these periods. We have relaxed the window forward in order to be symmetric with the relaxation of time before and after the symptoms by using 8 days after symptoms and find that the results do not change. This is expected since patients are expected to be either at home in quarantine or in hospital, in average, 5.5 days (95% CI 4.6 - 6.6 d) after first symptoms, according to [17]. Indeed, we find that the displacements of patients are highly reduced after the days of first symptoms. Thus, we expect that the majority of contacts are established before symptoms, highlighting the necessity of contact tracing back in time.

S7.8 Unmatched cases

There are uncertainties in the number of cases matched between datasets due to the fact that only a fraction of patient cases can be matched to the GPS dataset. The unmatched cases cannot be matched at random to complete the data since these would ignore the correlations between the disease and behaviour. Thus, we do not consider the unmatched cases in the contact network. This unmatching is due to the incomplete coverage of the GPS dataset respect to the

real population. However, we have checked in Section S7.2 that the sampling coverage of the dataset is consistent with the real population which then minimizes the chances of small sampling bias. The spreading rate in our dataset is 0.112 as described in the Methods Section Model Calibration. This value corresponds to $R_0^{\text{cal}} = 2.66$ consistent with the real R_0 value in the whole population.

S7.9 Sparseness of the dataset

Due to the sparseness of the GPS data, we do not have access to all the contact between the infected people. To account for the smaller coverage of the GPS data in the calculation of R_0, we first obtain an effective $R_0' = 0.112$ valid for the smaller GPS dataset. This number obtained for the GPS dataset should be rescaled by the population ratio between the real population and the GPS sample, which is a factor of 23.77 and provides $R_0 = 2.66$, which is consistent with the values directly measured from the data.

It is important to note that this rescaling is only used to estimate the epidemiological parameter R_0 and does not in principle affect the further modeling of the contact networks, except for the fact that we use this estimated value R_0' to estimate the hyperparameters of the model (T, r). This means that, using other GPS datasets with different coverage, a new set of parameters needs to be determined to fine tune the model to the particular coverage of the GPS dataset used. Therefore, in a less sparser dataset in an actual application with larger number of mobile app users, the parameters of the model (T, r, p_c) should be calibrated accordingly.

We note that in our rescaling, we do not rescale the network, nor the in- or out-degree distributions, but just use the smaller GPS sample to obtain the hyperparameters of the model.

S7.10 Time-evolving weak ties

Weak ties in temporal networks have been investigated in [18]. In our case these weak ties are evolving with time as well. While we apply our definitions of centrality metrics to static networks defined over 15 days, we employ a moving window that calculates a new network every three days, thus extending the static definition of weak ties to temporal data.

S7.11 Infected cases

We have based our modeling on the evolving number of new cases, which is not as robust estimate as the number of death cases. The main indicator in contact tracing is the date of first symptoms. In the absence of this datapoint, the day of hospitalization can be also used to estimate the date of first symptoms from the hospitalization data using the ensemble average of the time interval from first symptoms to hospitalization across the rest of the patients. While death is a more accurate metric than these two metrics, an estimate of the day of first symptoms is more difficult to obtained from the date of death, which anyways, occurs to only a fraction of the patients.

S7.12 Contact tracing methods

In a contact tracing app based on GPS technology, a centralized server is needed where one entity has access to the GPS data. The algorithms discussed here are also feasible in contact tracing platforms using Bluetooth technology, as long as the detected neighbours are shared at a given point by a central server. This is because, the optimal tracing strategy requires the contacts of the contacts to build the network. On the other hand, the geolocalization of the red zone of contacts in a geographical map with precise locations shown in Figs 2 and 3, requires the use of GPS data and cannot be performed with Bluetooth-based technology.

S7.13 K-core infection

We mention an important point about the definition of the k-cores. Given a network, the same k-core can be composed of several disconnected components. This is what we see from Figs 4C and 4D, or for instance, the example 3-core in S3C Fig contains several disconnected components. Our analyses (displayed in Figs 4C and 4D) show that these disconnected components are connected by weak links, which if removed, may isolate the spreading of the virus inside one of the disconnected components of a k-core, containing the spreading of the pandemic to inside the disconnected component of the k-core. In other words, if a part of the k-core is infected, the disease will be controlled within a small group of that k-core and not extended to the rest of the network, if the affected component of the k-core is isolated from the rest by the removal of the weak link.

S7.14 Quarantines

It is plausible that the slow decay of cases from the infection peaks after lockdowns observed in many countries might be due, in part, to the lack of deployment of quarantines based on optimization principles. Our results could help to provide insight into the persistence of infection in many places.

S7.15 Model of infection

In our model, a transmission probability is calculated for a contact, which is then taken to be infectious if the probability is above a threshold. To avoid overestimating the importance of strong contacts we then define the recursive probability Eq. (1), $P_i[n]$, where one strong contact between two highly connected groups is not more important than many weak contacts. This regularization is highly efficient in converting the simple hit probability $p_i[n]$ which is observed to be not a good separator of a contact versus a non-contact since its distribution is not bimodal

(orange curve) as observed in S1A Fig, into a bimodal distribution for $P_i[n]$ as observed in the figure (blue curve). Thus, $P_i[n]$, which takes into account the importance of many small contacts separates well the contacts, and does not need of a precise threshold to be implemented. In fact, any threshold in the range $0.1 < p_c < 0.9$ gives similar results in the contact model since most of the cases are concentrated in the extreme cases $P_i[n] \approx 1$ and $P_i[n] \ll 1$ or zero. Thus, while the thresholding of $p_i[n]$ has a danger of throwing out many weak links, the use of $P_i[n]$ regularize this function in the proper way such that the effect of choosing a threshold is minimized. That is, since the distribution of $P(P_i[n])$ (S1A Fig) is highly bimodal concentrating near zero and one, then we were able to separate well a contact $P_i[n] \approx 1$ from a non-contact $P_i[n] = 0$, and the need for a threshold to distinguish between these two extremes disappears, since the probability is nearly zero between these two extreme values as shown in the figure.

S7.16 Dynamics of weak links

In principle, the networks are calculated continuously in time, and therefore the weak links are identified as they form and break k-cores. If by removing an individual, somebody else replaces that role, then a new network should identify this new individual added as a new weak link. However, this process may become somehow impractical due to the continuous removal of those weak links. This problem can be treated by determining the roles or occupations of the weak links and then search for a way to remove this risk by targeting those roles or occupations. This can be achieved by the analysis shown in, for instance, Figs 3C and 3D, by geolocalizing the weak links and k-core in the map to discover the occupations/roles or places visited by the weak links and develop a targeted approach accordingly. For instance, Fig 3D shows where these occupations and roles contributing to the transmission chain are occurring. Targeting these occupations and places to remove the risk is an efficient way to break the chain of transmission.

S7.17 Uncertainties in infected population

We match the infected individual by a rule that uses the geolocation of the individual at the address of the patient. This method may cause some uncertainty in the results as some fraction of the infected individuals could be in principle replaced by non-infected individuals. To study this problem, we test that the identified mobile ID has also visited the hospital and or a pharmacy on the date of test results. Using this second test, we corroborated the matching between patients and mobile users. See Section S7.3.

S7.18 Level of uncertainty required for implementation

The methods of the algorithm requiere a level of certainty in the dataset that may be not appropriate for the level of uncertainty inherent in the underlying data. The reality of tackling SARS-CoV-2 with digital traces is that data incompleteness may not permit the type of analysis described here. If the data has a large uncertainty, using global network measures such as betweenness to break up the transmission network may not be practicable. To address whether the conclusions are robust we have undertaken study of bias and uncertainty. We perform a treatment of bias, uncertainty and data incompleteness by studying sampling bias in demographic variables like geographical population coverage, socio-economic status, age and gender.

Extensive analysis are reported in Section S7.2, **Sampling bias**, showing that the distributions of location, economic status, age and gender are similar under two-sample KS test to the distributions of the real data. That is, we cannot reject the hypothesis that the real data and the GPS data comes from the same distribution. Thus, our results suggest that a collection of apps with GPS geolocalization provides a statistically significant sample to study the behaviour of the real population.

Regarding the use of global quantities, such as the betweeness centrality, it may not seen

practical, in principle, due to the necessity to obtain the global network. However, according to the Covid Tracing Tracker from MIT Technology Review at `https://bit.ly/2Y1NMet`, which tracks the contact tracing apps around the world, 35% of government-backed contact tracing apps are based on the same GPS technology used in our study and are able to provide the network of contacts needed for our algorithm to work. These GPS-based apps capture the necessary information on the global contact network needed to perform the present analysis, and our algorithm can be directly applied to them.

S7.19 Global measures and scalability

A drawback for the use of a global measure like betweeness centrality is the poor scalability of the measure for large system sizes. However, there are linear approximations of these algorithms that can be used to approximate the metrics for large systems [13]. For larger datasets approximate fast algorithms can be used to calculated BC, see Ref. [13]. Furthermore, once the network is obtained, the chain of transmission can be destroyed by other measures other than BC, which scales linearly with system size and are quite fast to calculate like the degree or CI, although not as optimal as through the weak links as shown by our results.

S7.20 Asymptomatic cases

Detecting asymptomatic cases is one of the biggest challenge of the COVID-19 pandemic. We have included the existence of contacts with asymptomatic in our model. Our method allows, in principle, the determination of possible contacts with asymptomatic infectious people. As explained in Fig 1A and further explained in S7 Fig, we extend the exposure period to -14 days from the day of first symptoms. At the same time, the infectious period starts -2 days from symptoms according to [16]. Therefore the contacts identified between -2 days and -14 days (labeled as E' in Fig 1A and also as the inbound contacts in S7 Fig) correspond to

inbound exposures from asymptomatic carriers. Thus, our model treats asymptomatic cases by considering this exposure period and accounts for possible two-chains of infection as shown in S7 Fig. Since the exposure period with asymptomatic (-14 to -2 days) is longer than the period of infectiousness (-2 to +5 days), we obtain a larger number of asymptomatic exposures than infectious transmission contacts per patient.

Detecting asymptomatic is the key to stop this pandemic. Asymptomatic detected at exposure E' should be immediately tested, even though they have not presented symptoms. Beyond our modeling, we are not aware of other contact methods that have attempted to detect asymptomatic contacts (beyond large-scale widespread testing). Our contact tracing algorithms might be able to detect those asymptomatic transmissions which are critical to stop the pandemic.

S7.21 Tenuous contacts

Linking infected individuals to GPS traces via space is tenuous and multiple hits are more than likely. To address this problem we first investigated the correlation between a unique mobile ID and a unique patient as discussed in Section S7.3. Furthermore, to address the multiple hits that are likely to lead to contagion, we have used the recursive probability $P_i[n]$ defined in Eq. (1) and explained thereafter.

S7.22 Quantification of uncertainty in data

Quantification of uncertainty in data plotted in the figures is done via the calculation of the standard error (SD). For the SIR models, the errors are computed as the SE for a given value of the k-core. We notice that some plots are single instances, like for instance the giant connected component, and do not have SE.

S7.23 Privacy considerations

Ethics and privacy consideration of data sharing and using large datasets in biomedical research.

The patient datasets were collected by the Epidemiological Surveillance Department, Fortaleza Health Secretariat (CEVEPI) at the Prefeitura de Fortaleza, Ceará, Brazil by the team of Dr. Antonio S. Lima Neto. The present project follows the recommendations of the Wellcome Trust 2016 *"Statement on data sharing in public health emergencies"*, that states: *"In the context of a public health emergency of international concern, there is an imperative on all parties to make any information available that might have value in combating the crisis"* and the statement of the WHO: *"data are the basis for all sound public health actions"*. The Epidemiological Surveillance Department have provided the anonymized data on COVID-19 patients in the City of Fortaleza including the SARS-COV-2 test detection date and first day of symptoms of COVID-19 geocoded cases. Prior to be analyzed, the dataset was completely de-identified at CEVEPI. The data did not include any element that allow us to make a full profile of the patient, such as, the name of the patient, neither the residential address. Additionally, there were no codes associated with the variables that may allow individuals to be identified.

The policy statement for the use of the data states that the information was collected and anonymized by CEVEPI and analyzed by the team at CCNY and UFC, and then deleted completely from the servers of the team at both CCNY and UFC. At the time of submission, the provided datasets have already been deleted from the servers at CCNY and UFC. The data were made available to the team over a period from March 1, 2020 to June 7, 2020 for the purpose of helping the government contain the pandemic. The protocol of contact tracing developed in this study was done within the framework of the public functions of the government aimed at protecting and guaranteeing the public health of the citizens.

The team has signed confidentially agreements stating that the member of the team are

responsible for the custody of the data received and that we guarantee the privacy, the confidentiality, anonymization and used of the obtained information of the patients and any third party. The confidentially agreement also states that the team will receive the data only for the purpose of the current investigation and that all data will be erased from the servers at CCNY and UFC at the end of the project in July 2020. All data has been already deleted from the servers at CCNY and UFC and remains under the control of Department of Epidemiological Surveillance, Fortaleza Health Secretariat.

Given the extraordinary nature of this health emergency, it is critical for governments and agencies to share these data with scientists to execute critical studies under the understanding that by using the contact tracing algorithms developed in this study, we could help avoid more casualties during the pandemic. The patient dataset from the Health Department authorities in Fortaleza has the required ethics approval from the IRB at CCNY and UFC. Consent to use these data was given by the Mayor of Fortaleza and the Prefeitura of the City of Fortaleza. The original dataset is kept under the care of the Health Secretariat of Fortaleza.

References and Notes

[1] Grandata-United Nations Development Programme partnership to combat COVID-19 with data. Accessed 31 Jan 2022. Available from: `https://covid.grandata.com`.

[2] Newman MEJ. Spread of epidemic disease on networks. Phys. Rev. E. 2002;66:016128.

[3] Caldarelli G, Vespignani A. Large Scale Structure and Dynamics of Complex Networks: From Information Technology to Finance and Natural Science. (World Scientific Vol. 2, 2007).

[4] Pastor-Satorras R, Castellano C, Van Mieghem P, Vespignani A. Epidemic processes in complex networks. Rev. Mod. Phys. 2015;87:925-979.

[5] Albert R, Jeong H, Barabási AL. Error and attack tolerance in complex networks. Nature. 2000;406:378-382.

[6] Cohen R, Erez K, ben-Avraham D, Havlin S. Breakdown of the Internet under intentional attack. Phys. Rev. Lett. 2001;86:3682-3685.

[7] Kitsak M, Gallos LK, Havlin S, Liljeros F, Muchnik L, Stanley HE, et al. Identification of influential spreaders in complex networks. Nat. Phys. 2010;6:888-893.

[8] Morone F, Makse HA. Influence maximization in complex complex networks through optimal percolation. Nature. 2015;524:65-68.

[9] Friedkin NE, Theoretical foundations for centrality measures. Am. J. Sociol. 1991;96:1478-1504

[10] Freeman LC. Centrality in social networks: Conceptual clarification. Social Networks. 1979;1:215-239..

[11] Barthélemy M. Betweenness centrality in large complex networks. Eur. Phys. J. B 2004;38:163-168.

[12] Hébert-Dufresne L, Allard A, Young JG, Dubé LJ. Global efficiency of local immunization on complex networks. Sci. Rep. 2013;3:2171.

[13] K. Madduri, D. Ediger, K. Jiang, D. A. Bader, D. G. Chavarría-Miranda. A faster parallel algorithm and efficient multithreaded implementations for evaluating betweenness centrality on massive datasets. Third Workshop MTAAP (2009).

[14] Pei, S. & Makse, H. A. Spreading dynamics in complex networks. *J. Stat. Mech.* **12**, P12002 (2013).

[15] C. Buckee. Improving epidemic surveillance and response: big data is dead, long live big data. *The Lancet Digital Health* **2**, e218-e220 (2020).

[16] He X, Lau EH, Wu P, Deng X, Wang J, Hao X, et al. Temporal dynamics in viral shedding and transmissibility of COVID-19. Nat. Med. 2020;26:672-675.

[17] Sanche S, Lin YT, Xu C, Romero-Severson E, Hengartner N, Ke R. High contagiousness and rapid spread of severe acute respiratory syndrome coronavirus 2. Emerg. Infect. Dis. 2020;26:7.

[18] R. K. Pan, J. Saramäki. Path lengths, correlations, and centrality in temporal networks. Physical Review E, **84**, 016105 (2011).

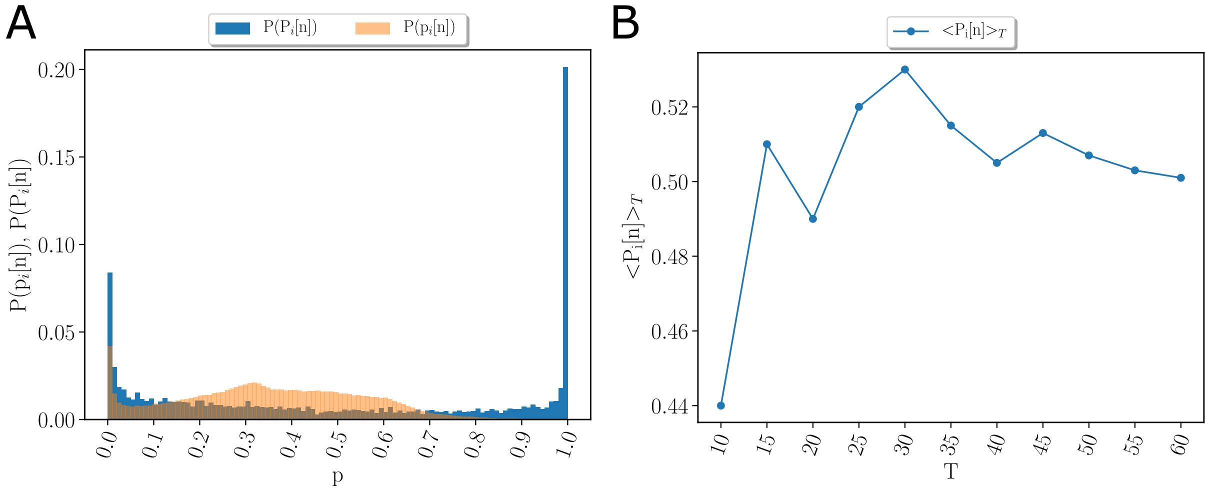

S1 Fig. Transmission probability. (A) Probability distribution of $p_i[n] = p_d[n] \cdot p_t[n]$ (orange) and the recursive form $P_i[n]$ defined in Eq. (1) (blue). The $P_i[n]$ are polarized to 0 and 1 becoming the best thresholded metric to use to consider a contact as infectious. **(B)** Average value $< P_i[n] >_T$ as a function of the time window T of the spatio-temporal contact area. $P_i[n]$ has a peak at T = 30 min; it decreases for $T > 30$ min and increase for $T < 30$ min as a function of T. The decreasing behaviour is what is expected, thus, 30 min is the minimum bound for the correct value of T.

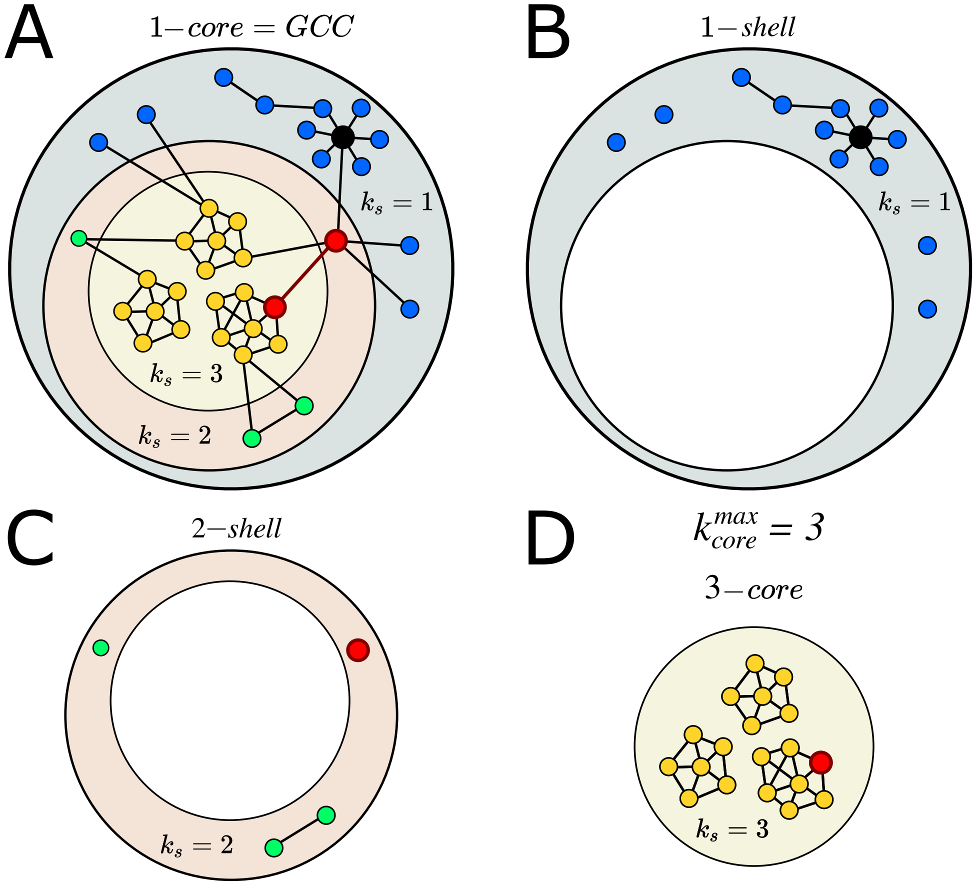

S2 Fig. Network structure under k-shell decomposition. **(A)** A sample network with 3 shells. The k-shell index k_s is not necessarily associated with other centralities. Here, the hub of the network in black with $k = 7$ is in the 1-shell, $k_s = 1$. The two top node in betweenness centrality, highlighted in red, belong to the 2-shell and the 3-shell, respectively. The 1-core is equivalent to the GCC. **(B)** The nodes with $k_s = 1$ form the 1-shell, **(C)** the nodes with $k_s = 2$ form the 2-shell, and **(D)** the nodes with $k_s = 3$ form the 3-shell which is also the 3-core.

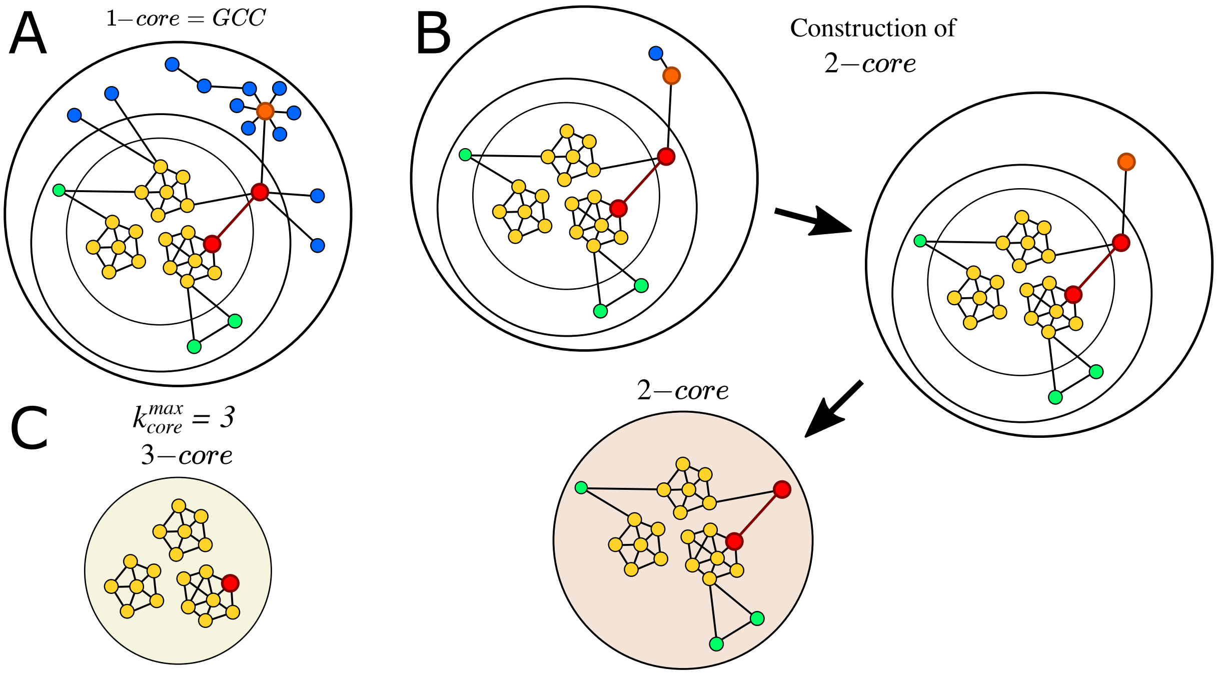

S3 Fig. K-cores of a network. **(A)** We start the k-shell decomposition with a network configuration where every node has at least degree $k = 1$. This set of nodes forms a 1-core. **(B)** Then, every node with $k = 1$ is iteratively removed to obtain the 2-core. As one can see, the removal of these nodes changes the degree distribution. Thus, nodes are removed until all remaining nodes are left with $k \geq 2$. **(C)** Following the k-shell decomposition nodes are removed until we obtain the 3-core. The 3-core can be made of multiple disconnected clusters.

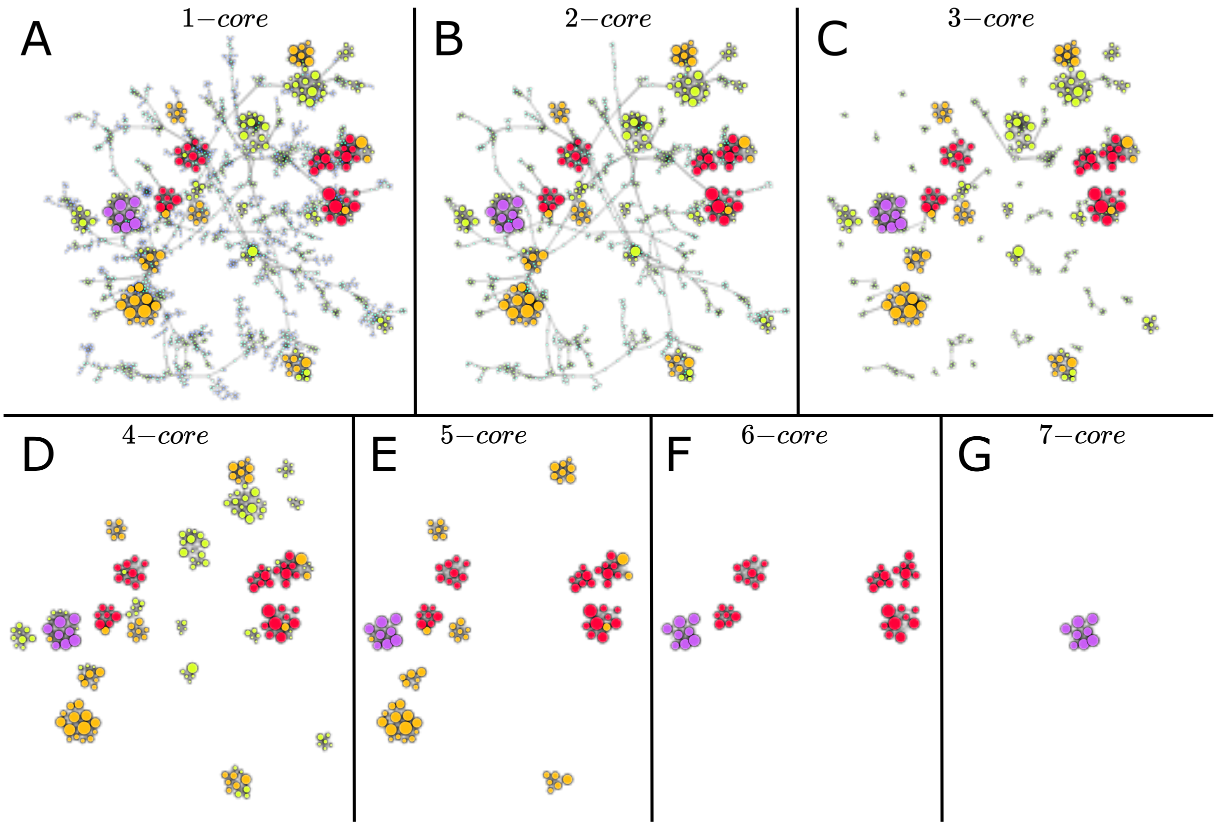

S4 Fig. K-cores decomposition. Example of k-core and k-shell structure in the network plotted in Fig 3B obtained during the lockdown. Here the colors are set by the k-shell occupancy of each node. Each k-core is composed by the k-shell plus the (k+1)-core. The k-cores are nested structures. For instance, the 5-core in **(E)** is composed by the 5-shell (yellow nodes) and the 6-core, which, in turn, is composed by the 6-shell (in red) and the 7-core (in purple). Since the 7-core is the maximal k-core, $k_{\rm core}^{\rm max} = 7$ for this network, then the 7-core is also the 7-shell. In this network the 0.5-core is the 4-core and the 0.5-shell is composed by the 1-shell plus the 2-shell and the 3-shell. We notice how a given k-core can be composed of many disconnected components. For instance, the 6-core is composed by 5 disconnected components. This is important, since each component of a given k-core can be localized in different areas, like different hospitals, in the map, see for instance, Figs 3C and 3D. It is also visually apparent that to destroy this network, a direct 'attack' to the high k-cores is not optimal. Instead, removing the high BC nodes that populate the lower k-shells is the best strategy. We plot each k-core in turn: **(A)** 1-core, **(B)** 2-core, **(C)** 3-core, **(D)** 4-core, **(E)** 5-core, **(F)** 6-core and **(G)** 7-core.

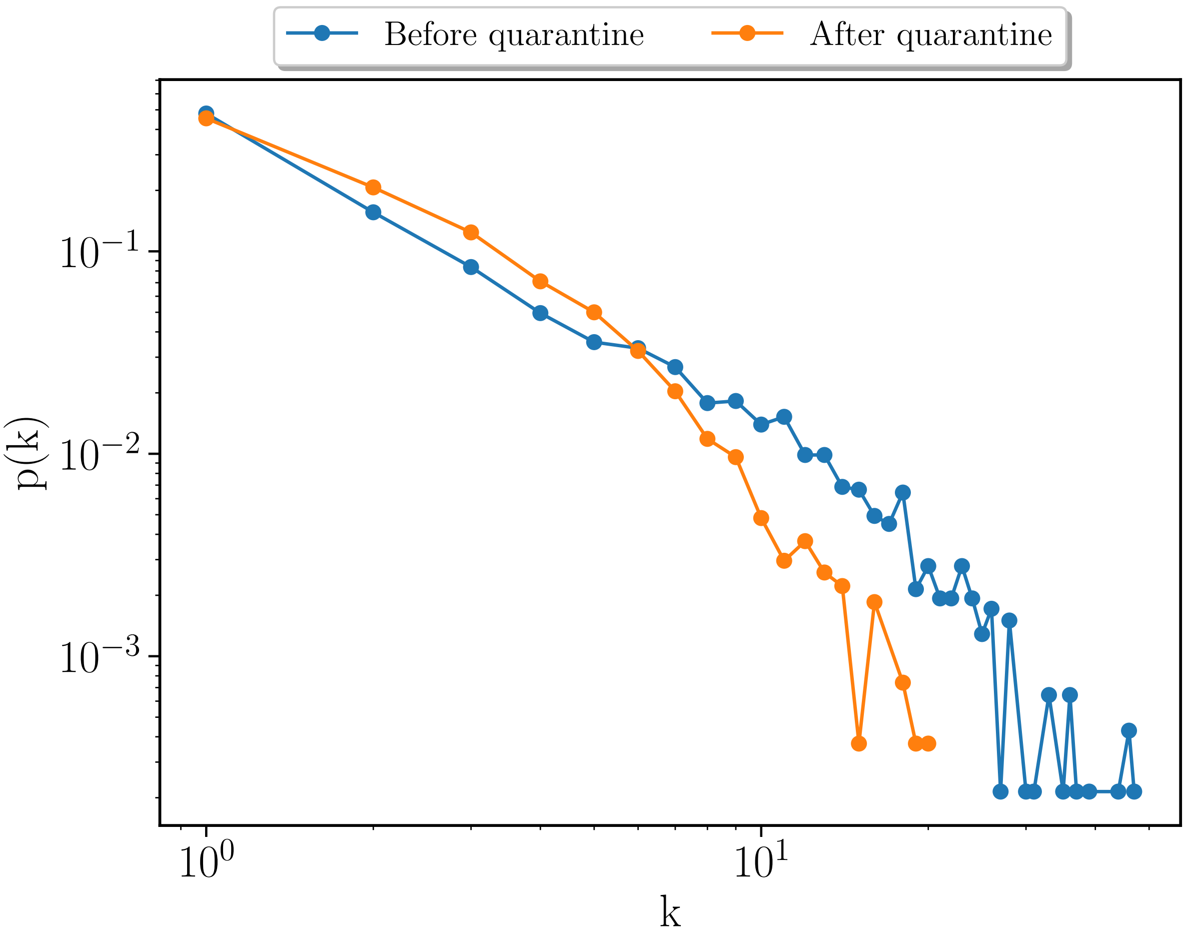

S5 Fig. Degree distribution of the contact network. Degree distribution of the contact network before (blue) and after (orange) the quarantine.

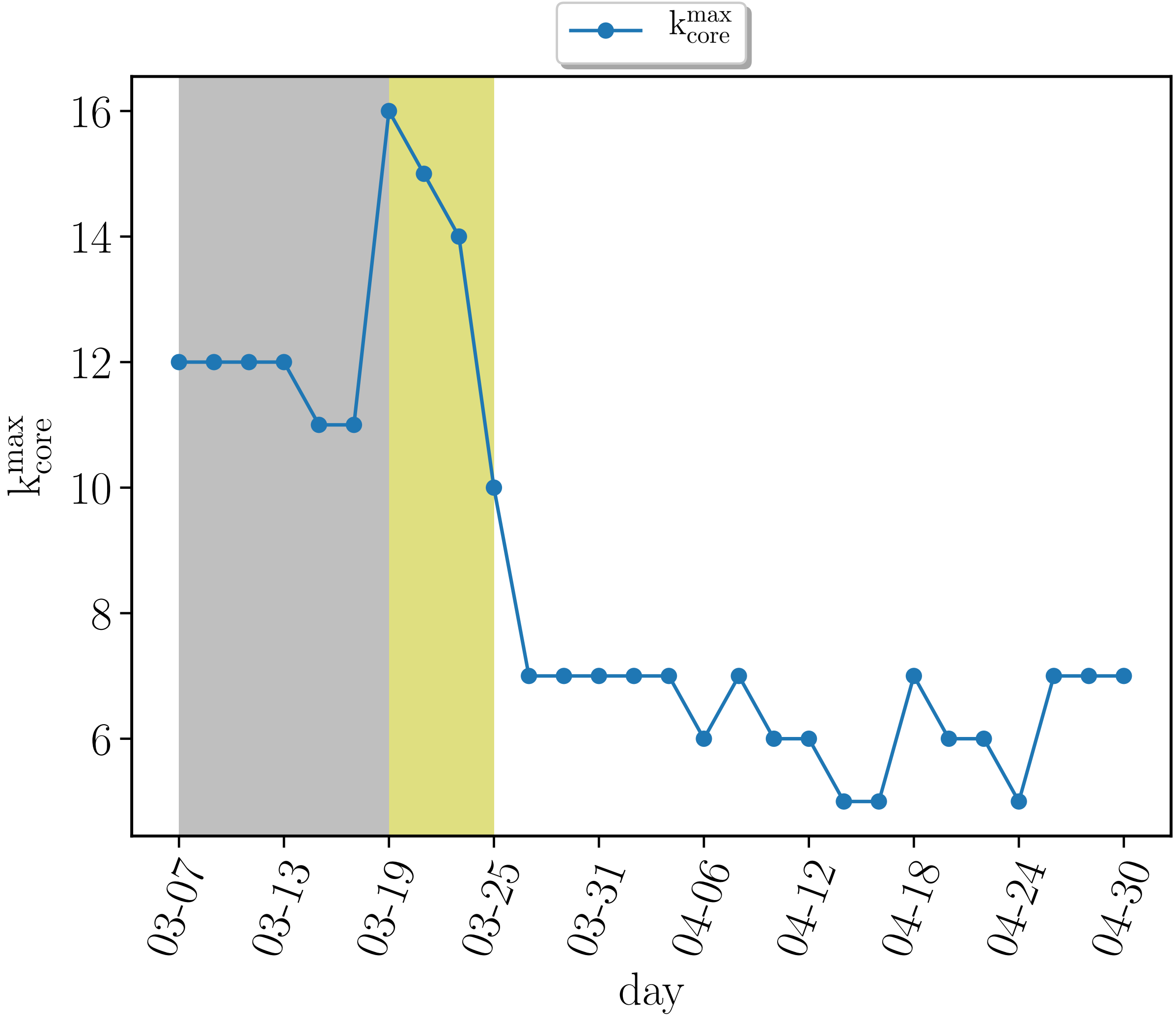

S6 Fig Evolution of the maximum k-core. Evolution of maximum k-core index k_{core}^{max} versus time previous to the quarantine (grey area), right after the quarantine (yellow area) and later. We see how the maximum k-core index drops drastically after the mass quarantine.

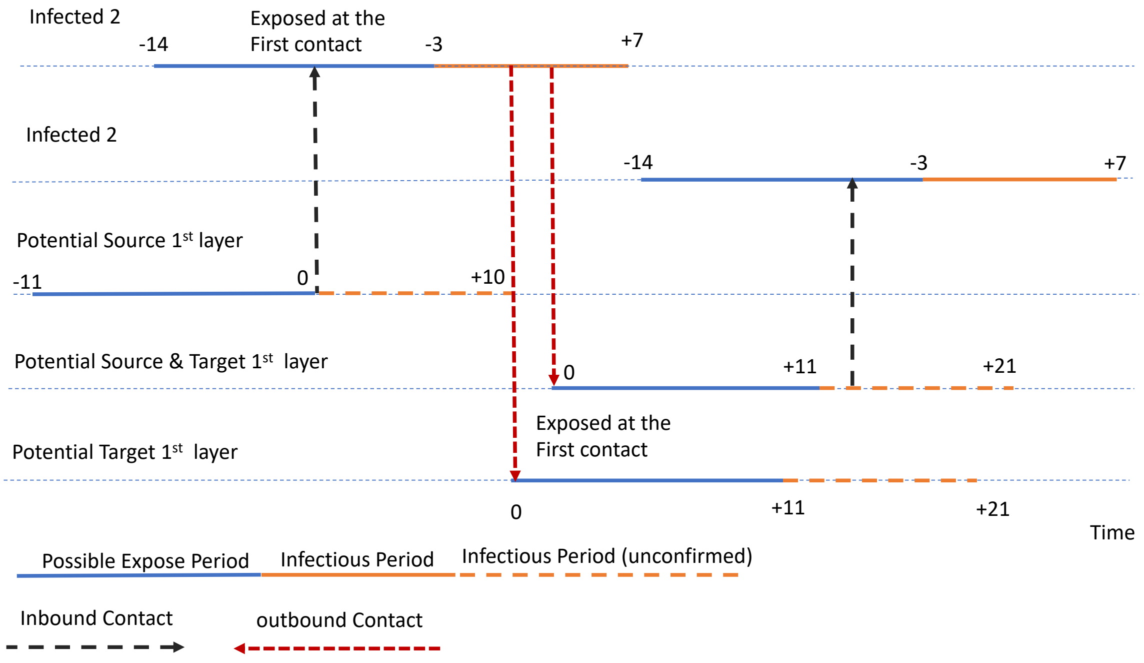

S7 Fig. Contact layers. Contact layers or pre-symptomatic and asymptomatic captured by the model. Our treatment of asymptomatic cases is to increase the exposure period to -14 days to accounting for possible two-chains of infection as shown in the figure. Contacts between -2 days to -14 days from the day of first symptoms are more likely to be an exposure from an asymptomatic infected person. Contact from -2 days to +7 days from first symptoms are considered to be transmissions contacts from the patient.

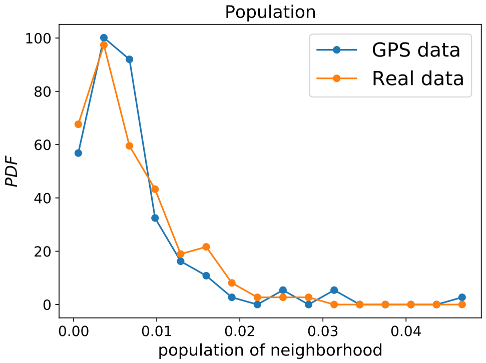

(A)

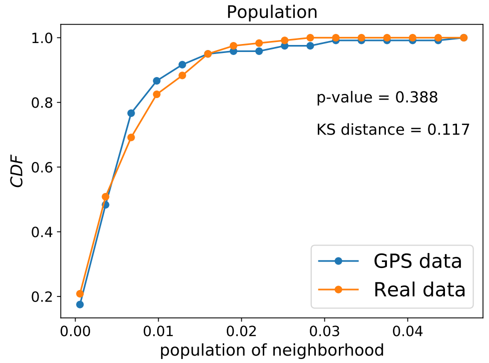

(B)

S8 Fig. Sampling bias-coverage. **(A)** Probability density function and **(B)** Cumulative distribution function of the fraction of the population per neighborhood in Fortaleza to the total population. We show the real distributions and the distributions from the apps GPS data. Both distributions pass a two-sample KS test indicating that we cannot reject the hypothesis that they come from the same distribution under the test.

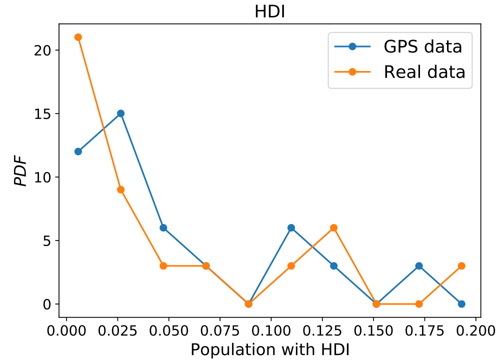

(A)

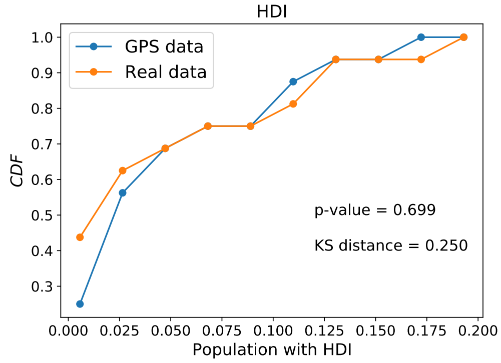

(B)

S9 Fig. Sampling bias-HDI. **(A)** Probability density function and **(B)** Cumulative distribution function of the fraction of the population per neighborhood with a given HDI in Fortaleza to the total population. We show the real distributions and the distributions from the apps GPS data. Two-sample KS test indicates that we cannot reject the hypothesis that the real and GPS sample come from the same distribution under the test, indicating lack of sampling bias under this test.

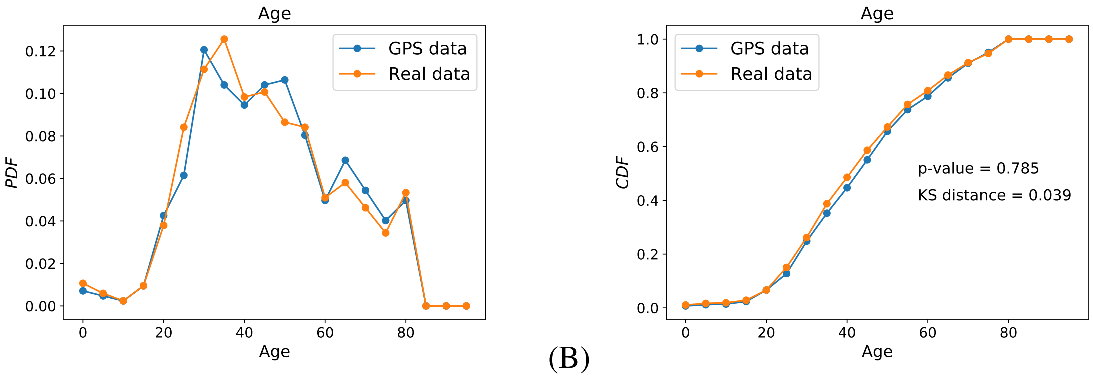

(A) (B)

S10 Fig. Sampling bias-age. **(A)** PDF and **(B)** CDF of age distribution in the GPS geolocalized data compared with the real patient data. We cannot reject the hypothesis that both samples come from the same distribution under KS statistical testing.

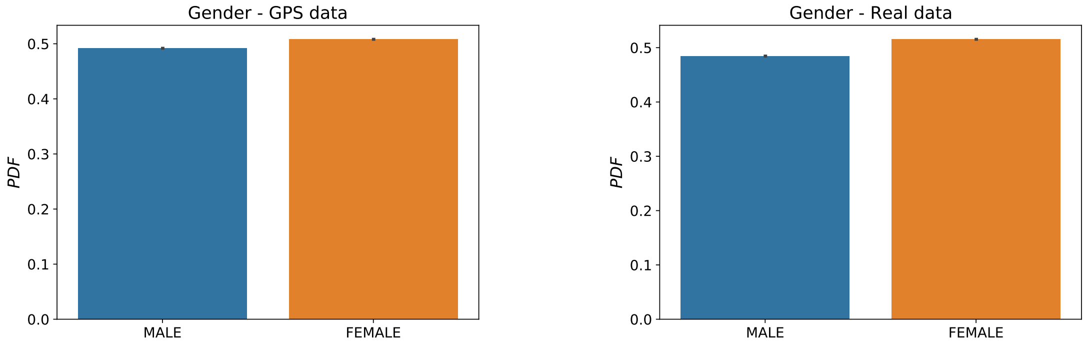

(A) (B)

S11 Fig. Sampling bias-gender. **(A)** PDF and **(B)** CDF of gender distribution in the GPS geolocalized data compared with the real patient data suggesting lack of bias.

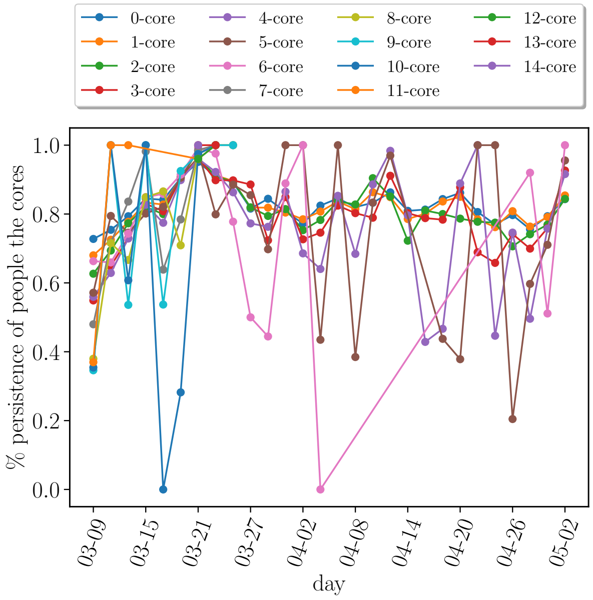

S12 Fig. K-core persistence. Persistence of people in the k-cores in the temporal networks. We plot the percentage of people in the cores from network to network. The persistance is calculated by the overlap of people in the k-shells from a time of observation to the next (three days later in this particular example).

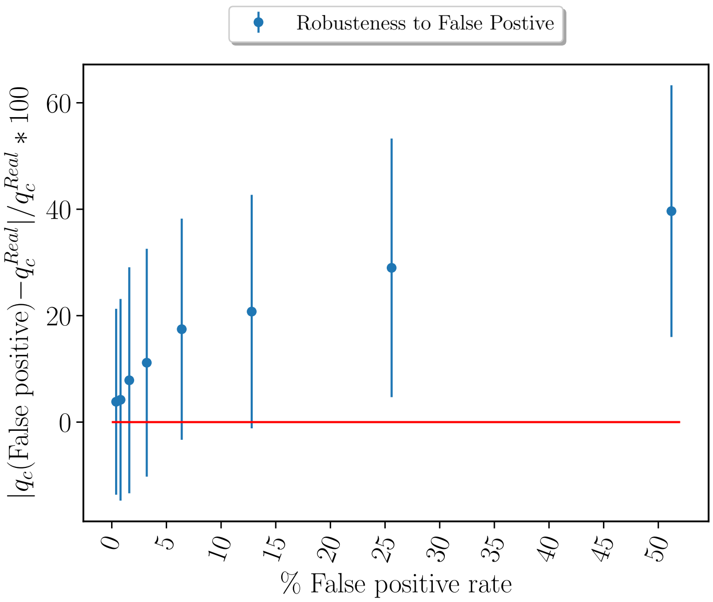

S13 Fig. Robustness to false positive. Normalized efficacy of BC centrality as a function of false positives in the report of infected people. A false positive is an individual who reported to have symptoms but was not infected with Covid-19. We plot the relative error in the determination of the minimal number of people to quarantine versus the false positive rate. The measure starts to deviate from linear behaviour beyond the error bars around 20% false positive rate.

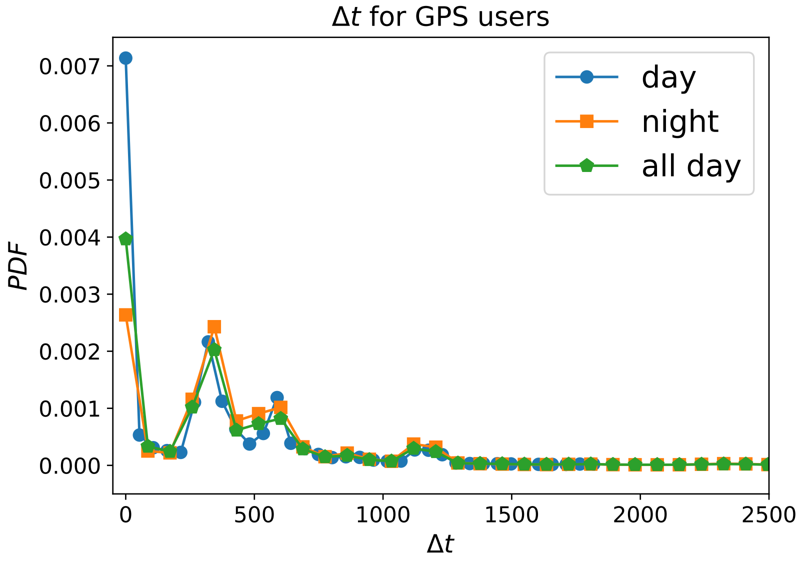

S14 Fig. GPS pings distribution. Distribution of the time interval between GPS pings during all day and separated by day and night.

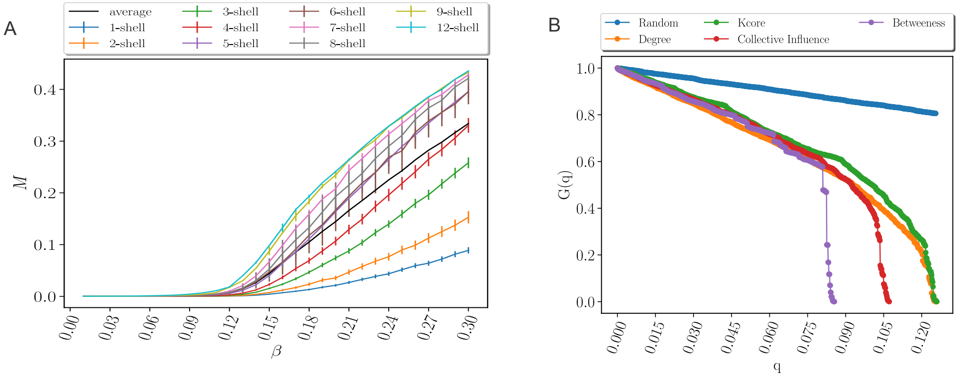

S15 Fig. Weak links and k-cores pre-quarantine. **(A)** Amount of infected population ($M = \sum \frac{M_i}{N}$ see [7]) when the spreading starts in a given node in a k-shell as a function of the probability of infection β for a SIR model on the same network on March 19 in Fig 3A in pre-quarantine Ceará. The black is the average value over all the starting nodes in the network. The average divides the shell contribution to the spreading of the virus in two groups above and below the average. The 0.5-core composed of the 6-core ($k_{\text{core}}^{\text{max}} = 12$ in this network) which contains nodes from the 6-shell to the 12-shell, has maximal spreading. The 0.5-shell which is composed by the remaining shell from 1-shell to 5-shell has minimal spreading, below the average. **(B)** Optimal percolation analysis performed over the network in Fig 3A before the quarantine on March 19 in Ceará with different attack strategies and their effect on the size of the largest connected component $G(q)$ versus the removal node fraction, q. Depending on the strategy nodes are removed: randomly (blue), by the highest value of betweenness centrality (green) [10, 9], degree (orange), collective influence (red) [8], and by the highest k-shell followed by high degree inside the k-shell [7]. After each removal we re-compute all the metrics. The best strategy among those studied is removing the nodes directly by the highest value of betweenness centrality.

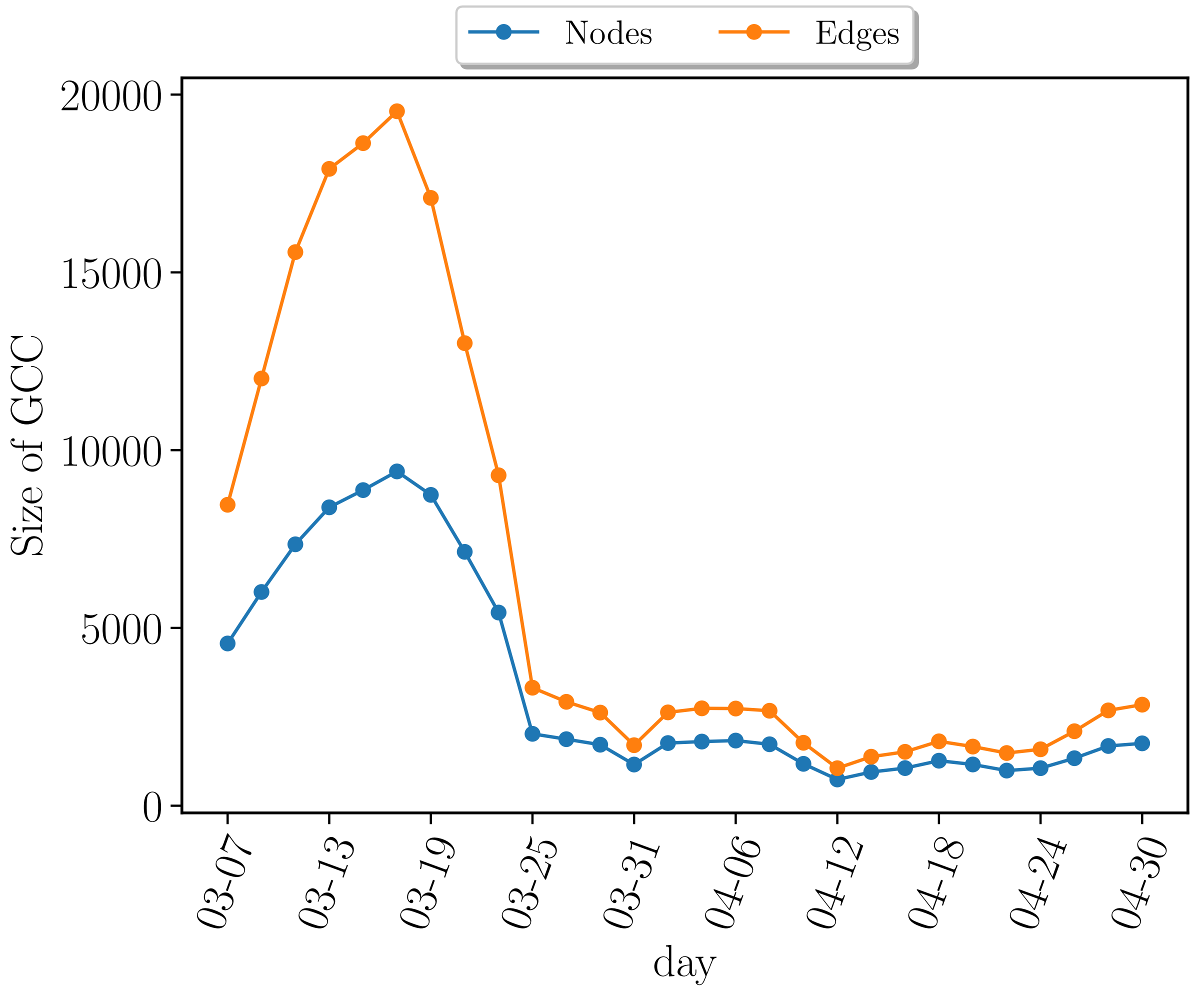

S16 Fig. Size of the GCC over time. The number of nodes (blue) and edges (oranges) in the GCC versus time. The initial increase in the number of nodes is artificial due to the fact that we perform contact tracing 14 days back for each patient and our data collection started in March 1. Thus the networks in the first two weeks have relatively lower contacts than the rest.

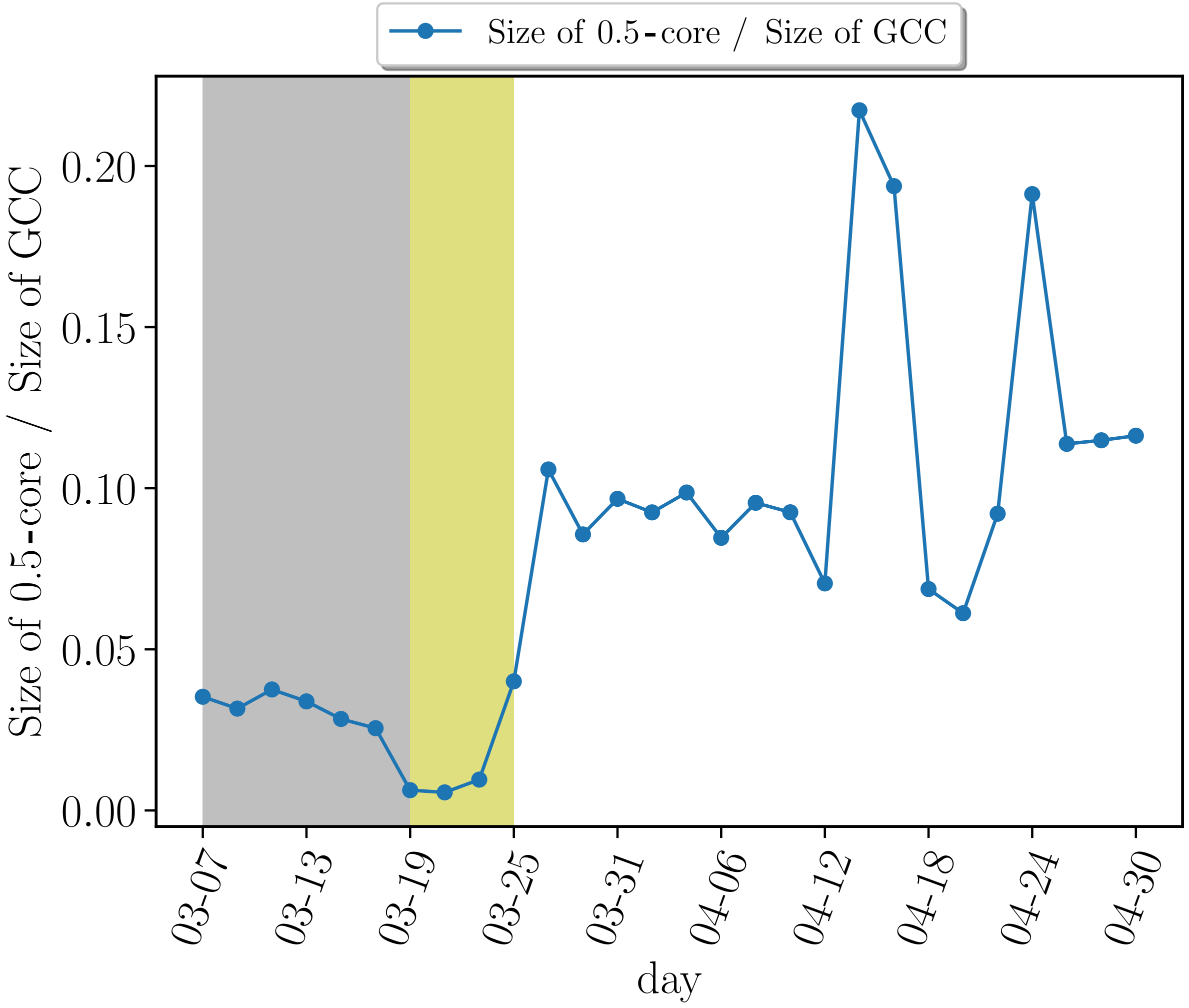

S17 Fig. Size of the 0.5-core over time. Evolution of maximum 0.5-core size versus time normalized by the size of the GCC. The proportion of these maximum k-cores keeps increasing after the quarantine.
