# Supplementary figures and images for: Digital contact tracing and network theory to stop the spread of COVID-19 using big-data on human mobility geolocalization
Source: PLoS Comput Biol. 2022 Apr 11;18(4):e1009865. doi: 10.1371/journal.pcbi.1009865 (PMC9053778; doi:10.1371/journal.pcbi.1009865)

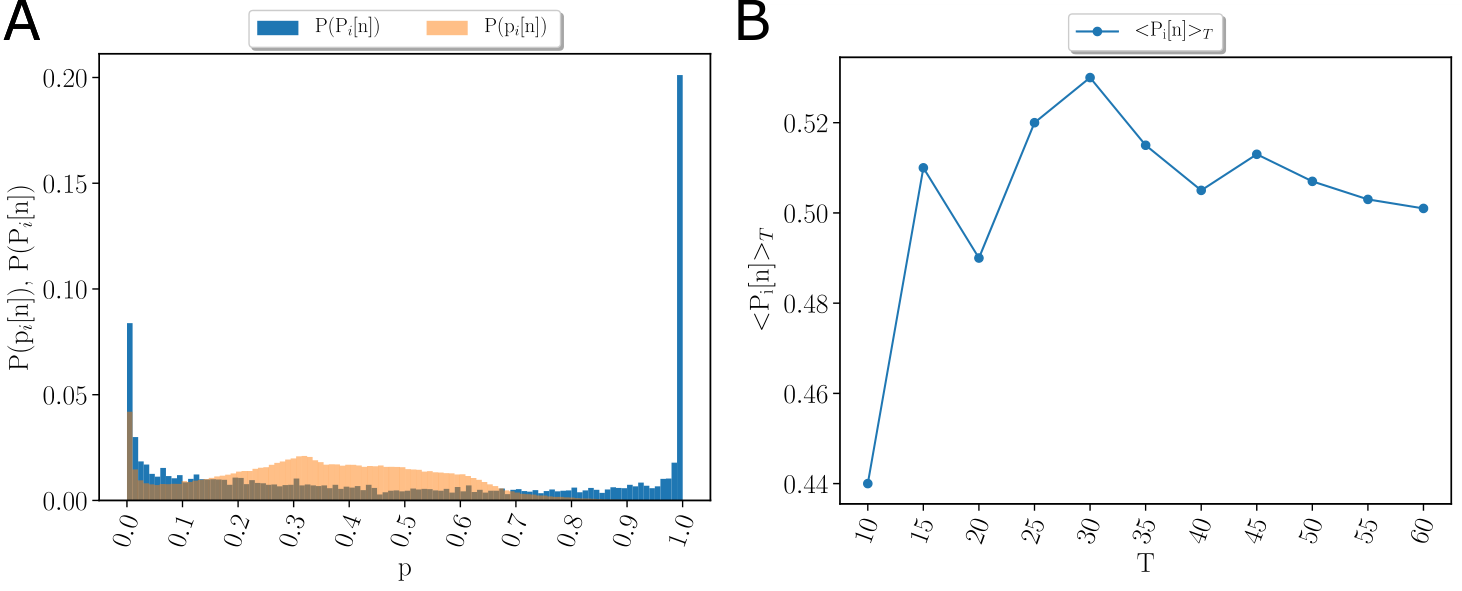

Supplement: S1 Fig — (A) Probability distribution of pi[n] = pd[n] ⋅ pt[n] (orange) and the recursive form Pi[n] defined in Eq (1) (blue). The Pi[n] are polarized to 0 and 1 becoming the best thresholded metric to use to consider a contact as infectious. (B) Average value < Pi[n] >T as a function of the time window T of the spatio-temporal contact area. Pi[n] has a peak at T = 30 min; it decreases for T > 30 min and increase for T < 30 min as a function of T. The decreasing behaviour is what is expected, thus, 30 min is the minimum bound for the correct value of T. (TIFF) [file pcbi.1009865.s002.tiff]

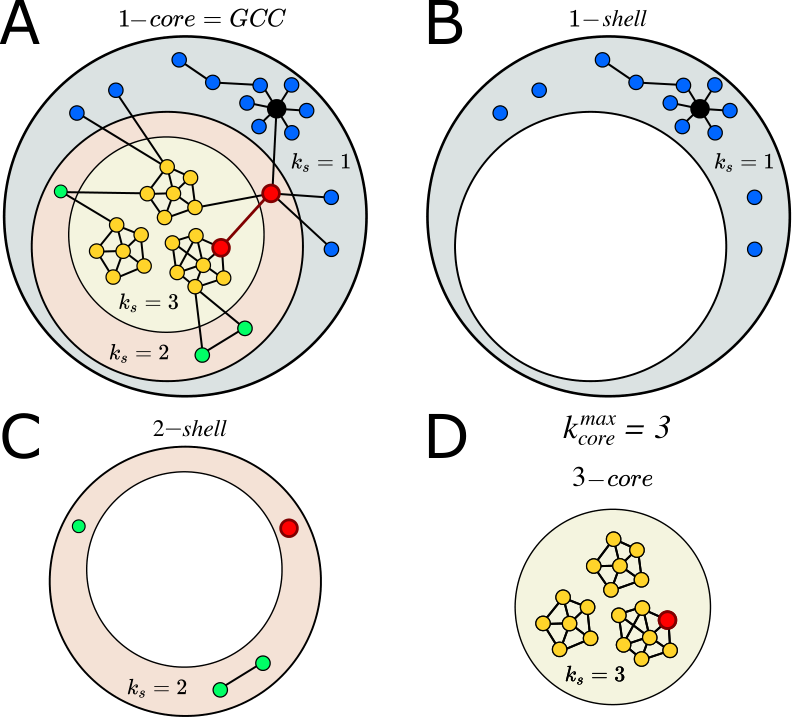

Supplement: S2 Fig — (A) A sample network with 3 shells. The k-shell index ks is not necessarily associated with other centralities. Here, the hub of the network in black with k = 7 is in the 1-shell, ks = 1. The two top node in betweenness centrality, highlighted in red, belong to the 2-shell and the 3-shell, respectively. The 1-core is equivalent to the GCC. (B) The nodes with ks = 1 form the 1-shell, (C) the nodes with ks = 2 form the 2-shell, and (D) the nodes with ks = 3 form the 3-shell which is also the 3-core. (TIFF) [file pcbi.1009865.s003.tiff]

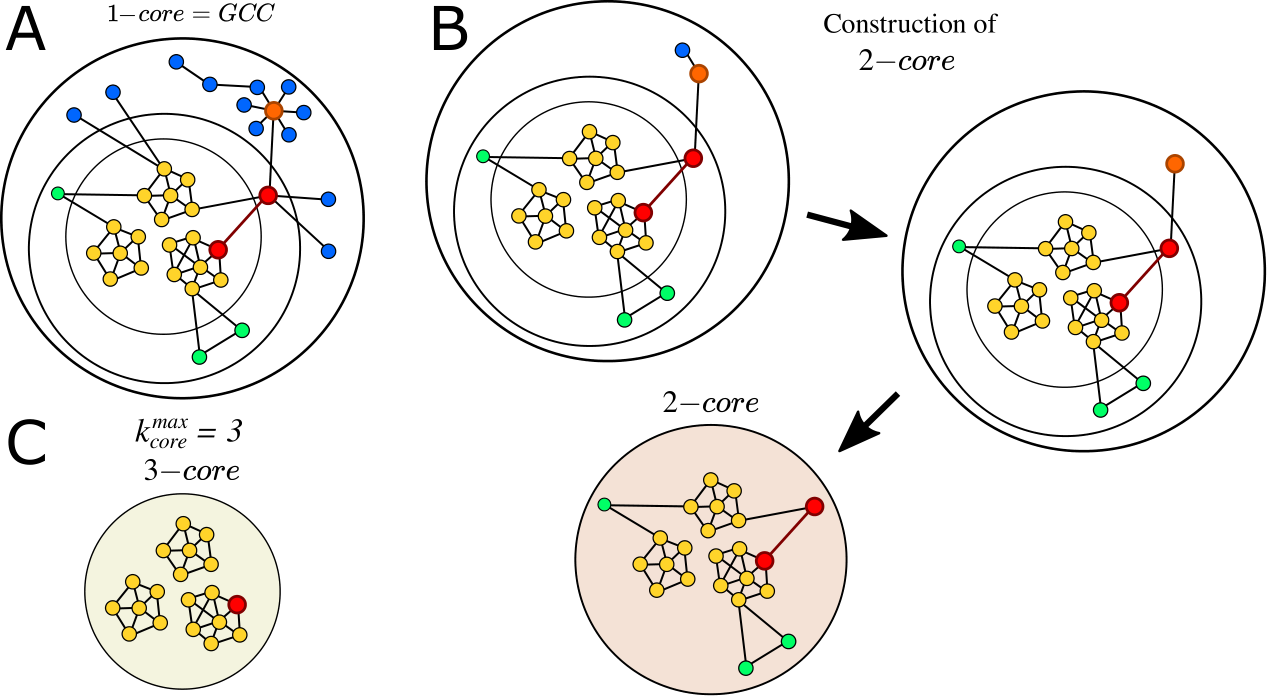

Supplement: S3 Fig — (A) We start the k-shell decomposition with a network configuration where every node has at least degree k = 1. This set of nodes forms a 1-core. (B) Then, every node with k = 1 is iteratively removed to obtain the 2-core. As one can see, the removal of these nodes changes the degree distribution. Thus, nodes are removed until all remaining nodes are left with k ≥ 2. (C) Following the k-shell decomposition nodes are removed until we obtain the 3-core. The 3-core can be made of multiple disconnected clusters. (TIFF) [file pcbi.1009865.s004.tiff]

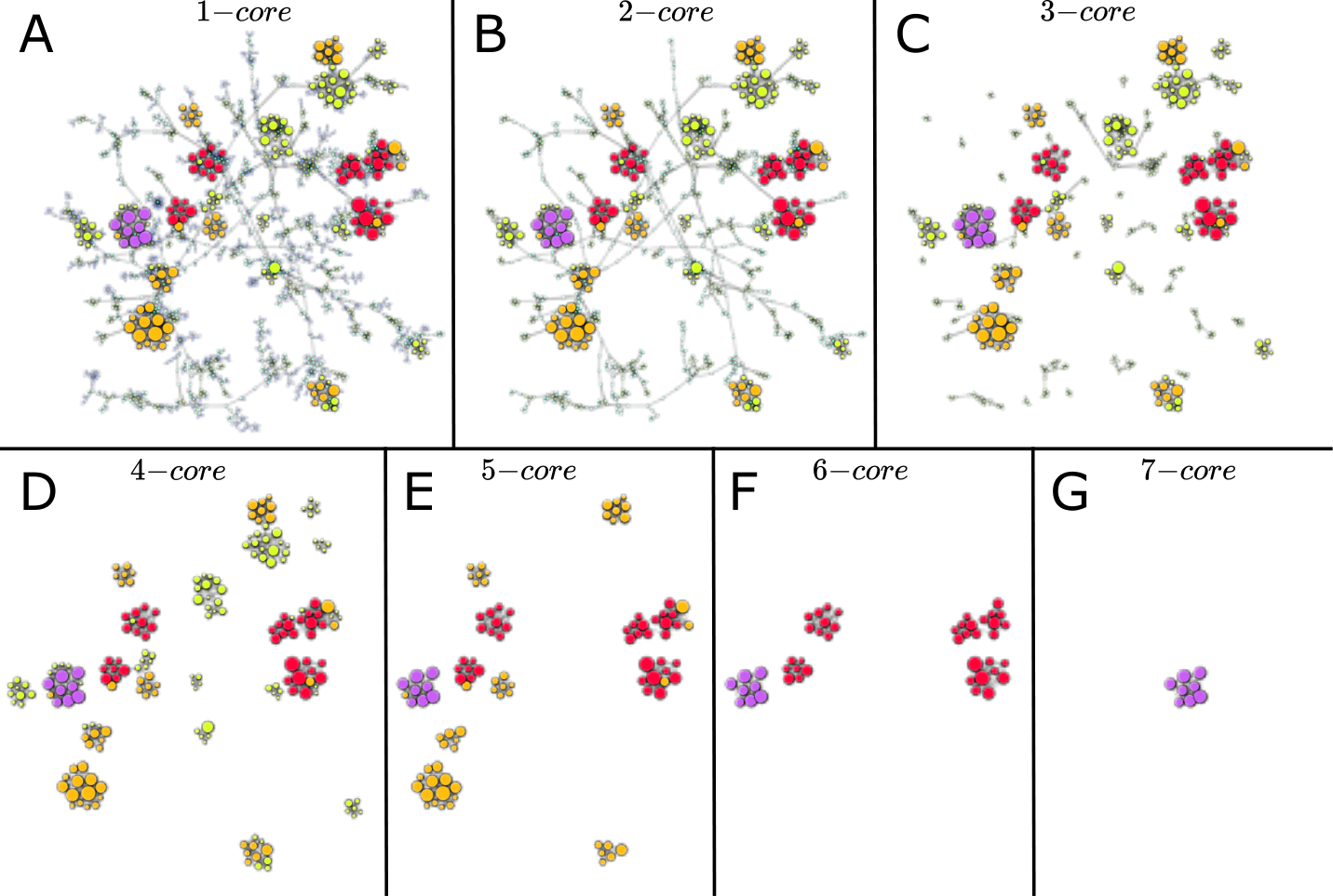

Supplement: S4 Fig — Example of k-core and k-shell structure in the network plotted in Fig 3B obtained during the lockdown. Here the colors are set by the k-shell occupancy of each node. Each k-core is composed by the k-shell plus the (k+1)-core. The k-cores are nested structures. For instance, the 5-core in (E) is composed by the 5-shell (yellow nodes) and the 6-core, which, in turn, is composed by the 6-shell (in red) and the 7-core (in purple). Since the 7-core is the maximal k-core, kcoremax=7 for this network, then the 7-core is also the 7-shell. In this network the 0.5-core is the 4-core and the 0.5-shell is composed by the 1-shell plus the 2-shell and the 3-shell. We notice how a given k-core can be composed of many disconnected components. For instance, the 6-core is composed by 5 disconnected components. This is important, since each component of a given k-core can be localized in different areas, like different hospitals, in the map, see for instance, Fig 3C and 3D. It is also visually apparent that to destroy this network, a direct ‘attack’ to the high k-cores is not optimal. Instead, removing the high BC nodes that populate the lower k-shells is the best strategy. We plot each k-core in turn: (A) 1-core, (B) 2-core, (C) 3-core, (D) 4-core, (E) 5-core, (F) 6-core and (G) 7-core. (TIFF) [file pcbi.1009865.s005.tiff]

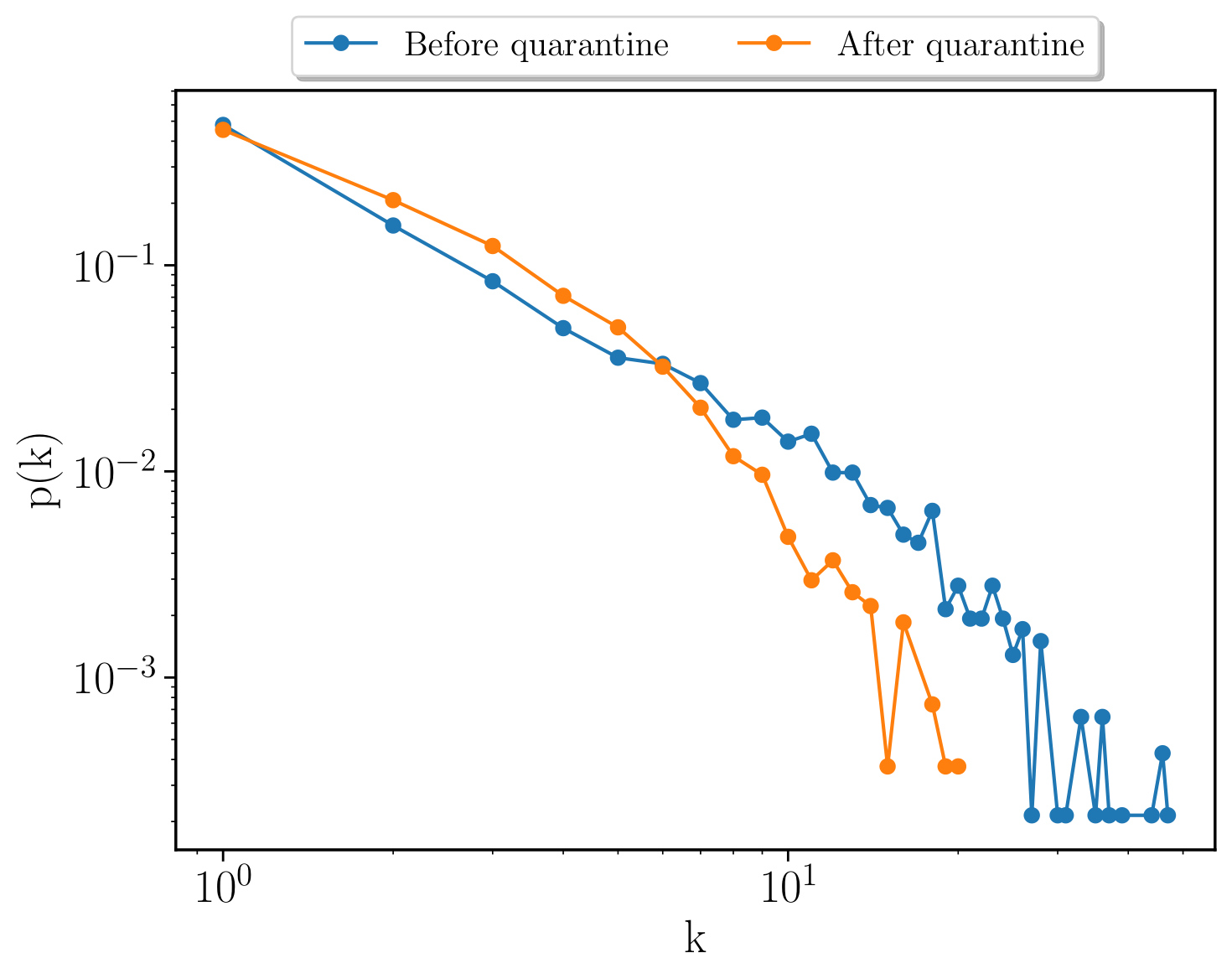

Supplement: S5 Fig — Degree distribution of the contact network before (blue) and after (orange) the quarantine. (TIFF) [file pcbi.1009865.s006.tiff]

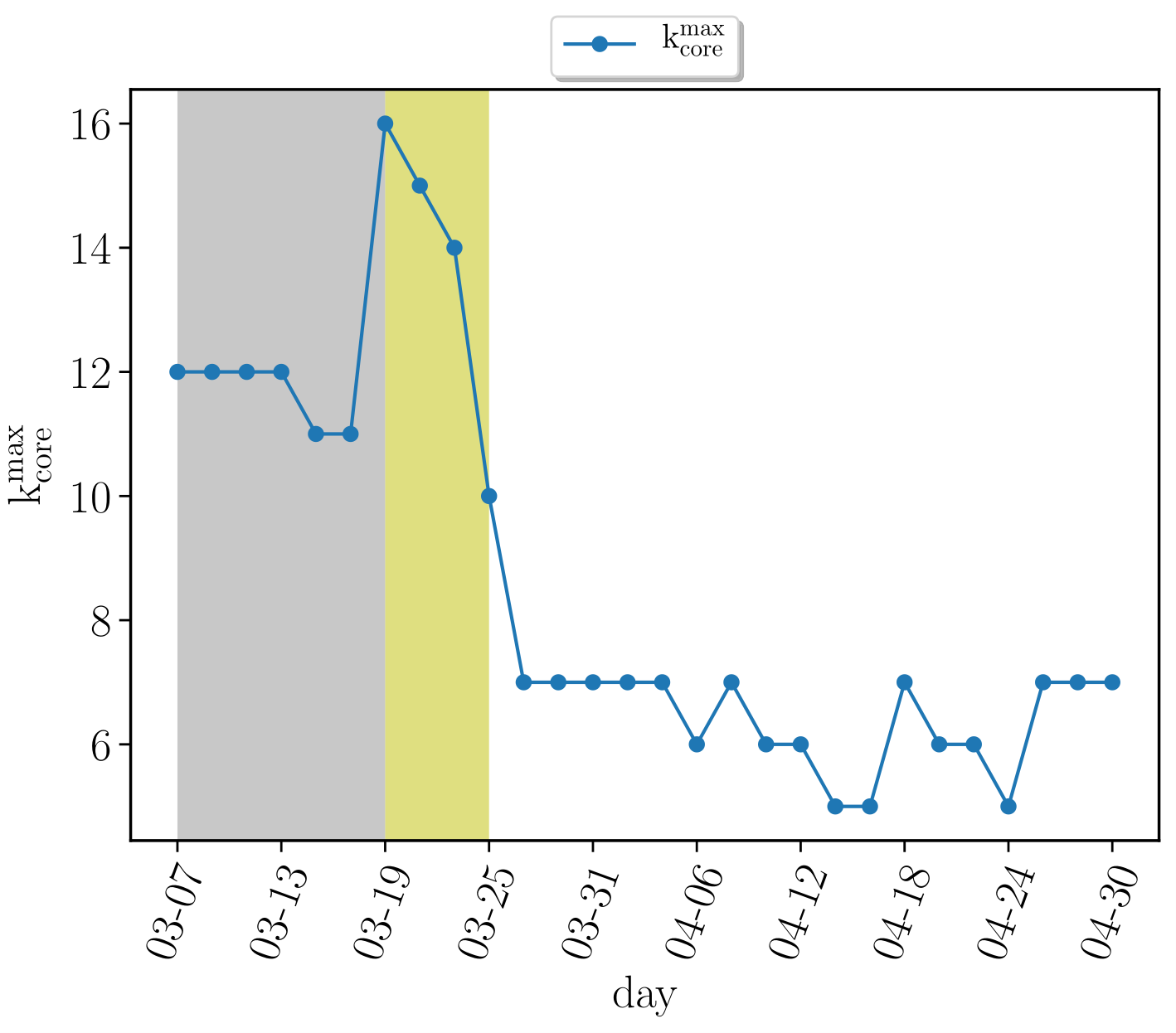

Supplement: S6 Fig — Evolution of maximum k-core index kcoremax versus time previous to the quarantine (grey area), right after the quarantine (yellow area) and later. We see how the maximum k-core index drops drastically after the mass quarantine. (TIFF) [file pcbi.1009865.s007.tiff]

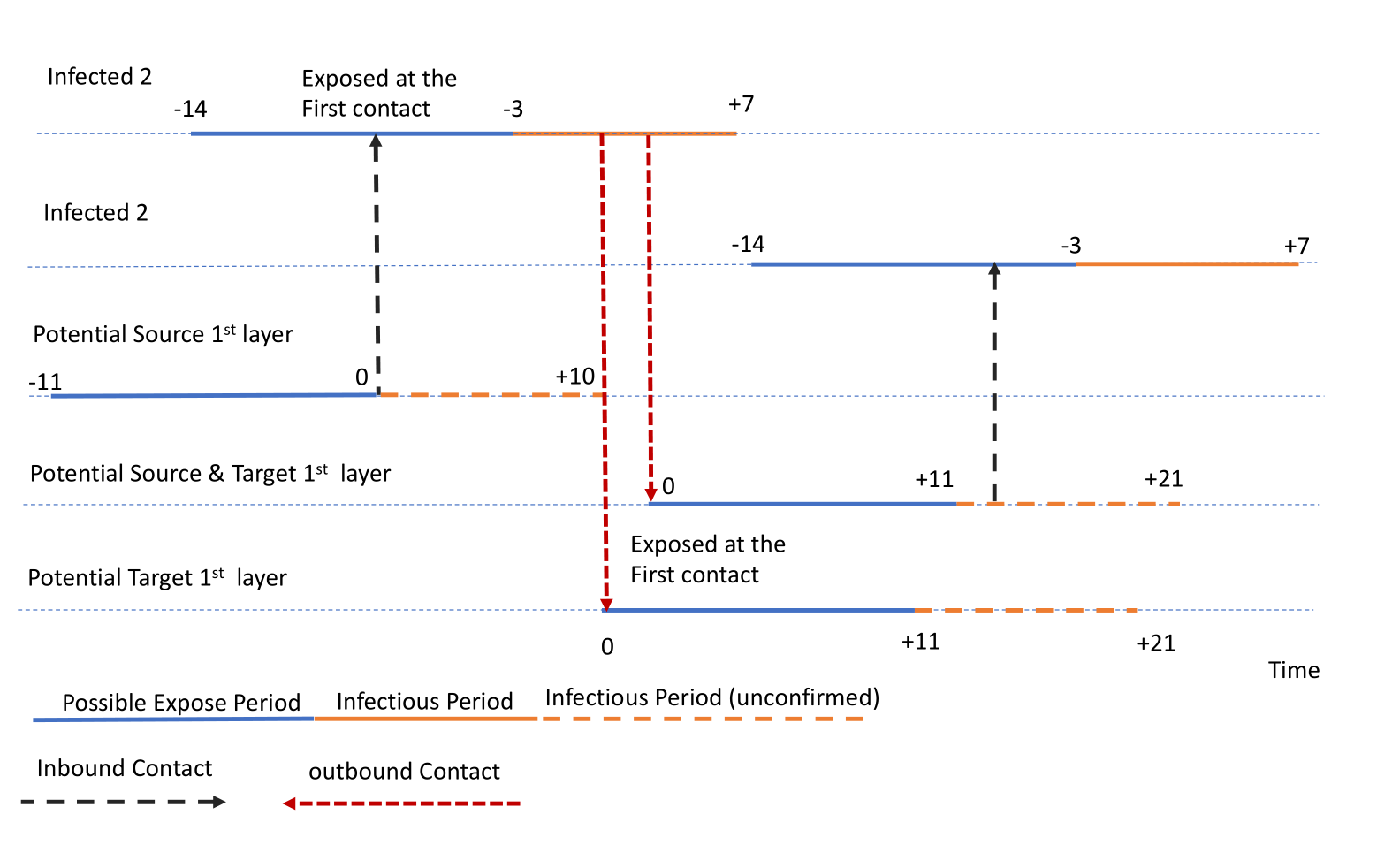

Supplement: S7 Fig — Contact layers or pre-symptomatic and asymptomatic captured by the model. Our treatment of asymptomatic cases is to increase the exposure period to -14 days to accounting for possible two-chains of infection as shown in the figure. Contacts between -2 days to -14 days from the day of first symptoms are more likely to be an exposure from an asymptomatic infected person. Contact from -2 days to +7 days from first symptoms are considered to be transmissions contacts from the patient. (TIFF) [file pcbi.1009865.s008.tiff]

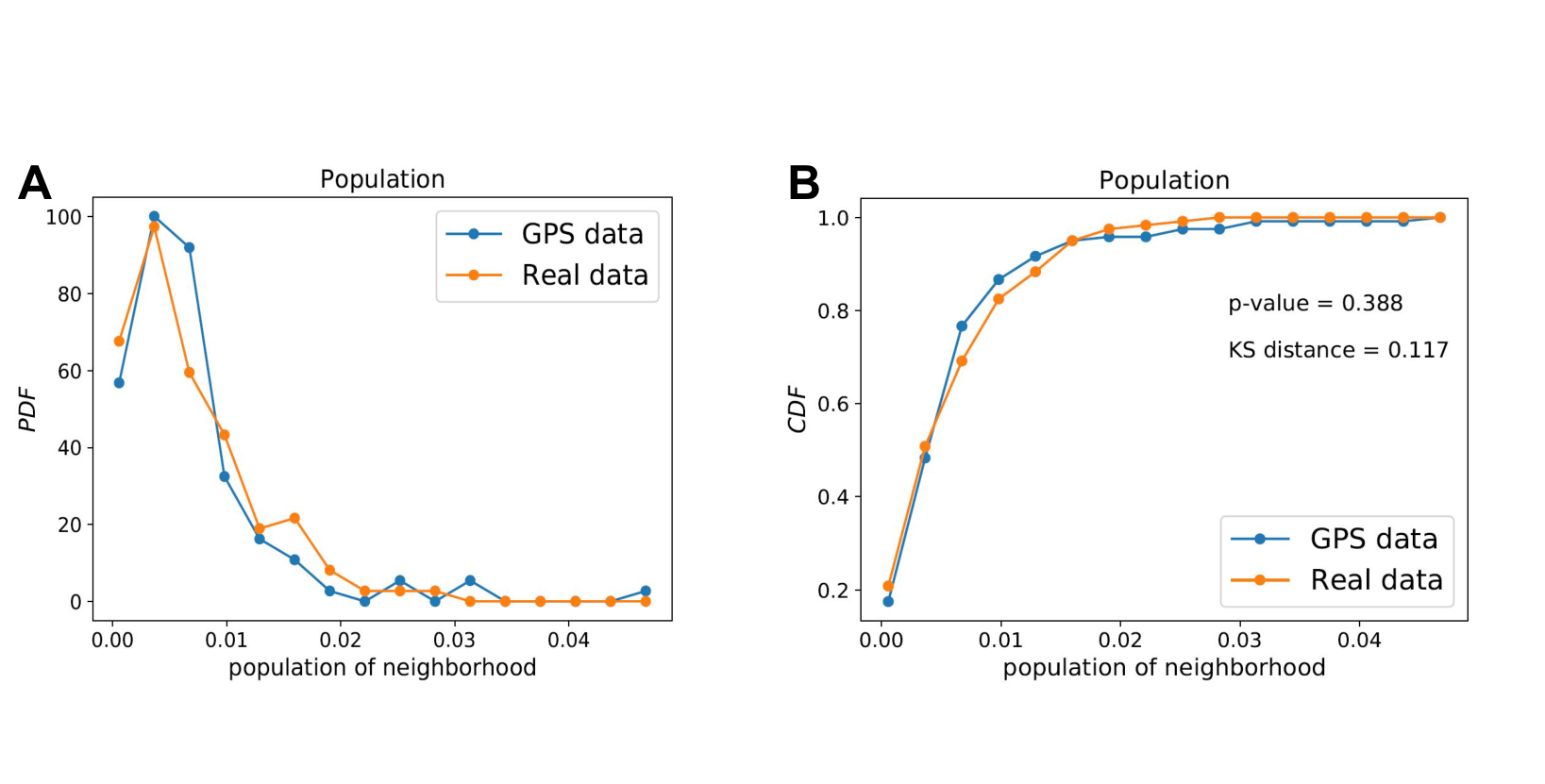

Supplement: S8 Fig — (A) Probability density function and (B) Cumulative distribution function of the fraction of the population per neighborhood in Fortaleza to the total population. We show the real distributions and the distributions from the apps GPS data. Both distributions pass a two-sample KS test indicating that we cannot reject the hypothesis that they come from the same distribution under the test. (TIFF) [file pcbi.1009865.s009.tiff]

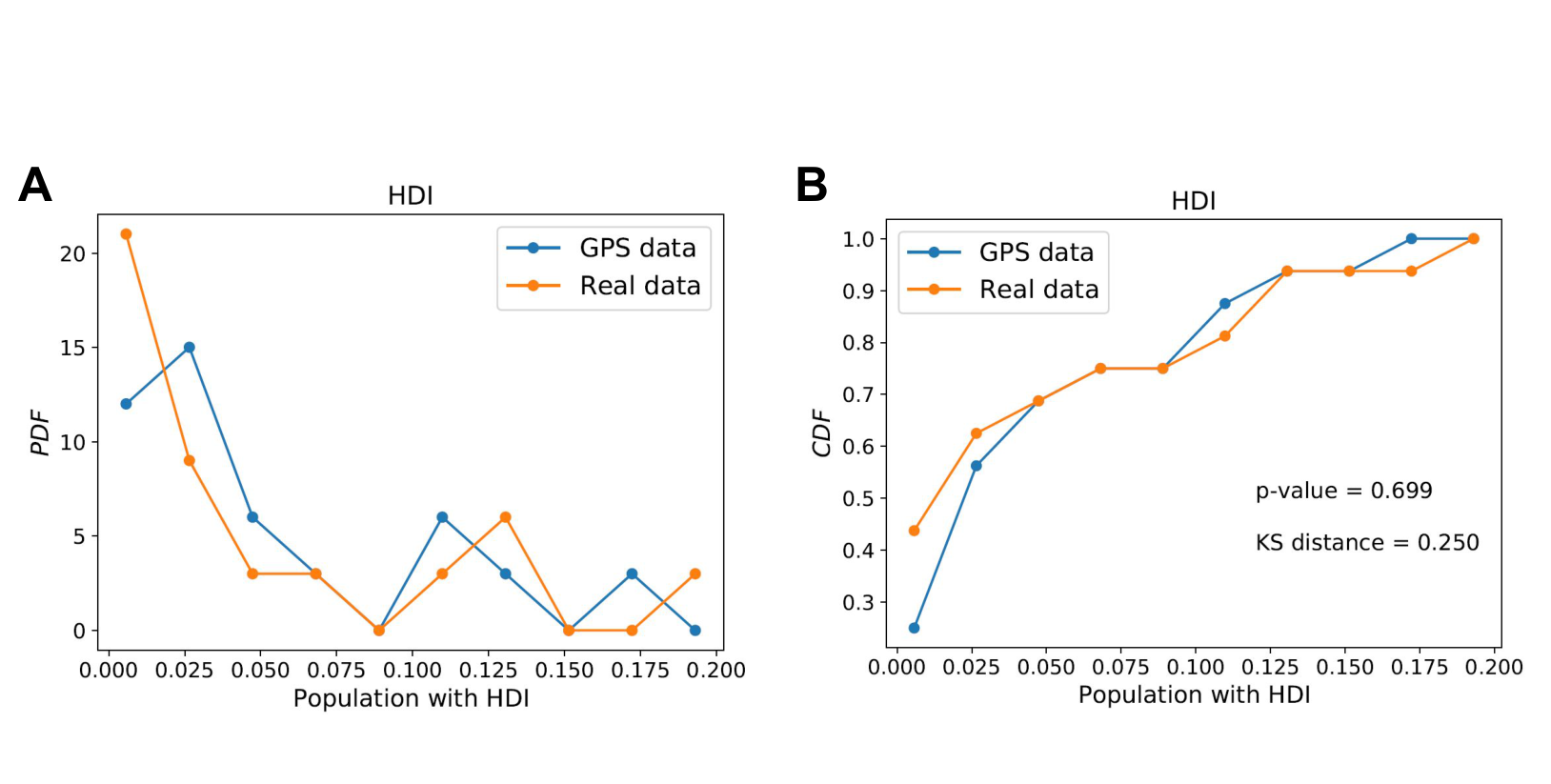

Supplement: S9 Fig — (A) Probability density function and (B) Cumulative distribution function of the fraction of the population per neighborhood with a given HDI in Fortaleza to the total population. We show the real distributions and the distributions from the apps GPS data. Two-sample KS test indicates that we cannot reject the hypothesis that the real and GPS sample come from the same distribution under the test, indicating lack of sampling bias under this test. (TIFF) [file pcbi.1009865.s010.tiff]

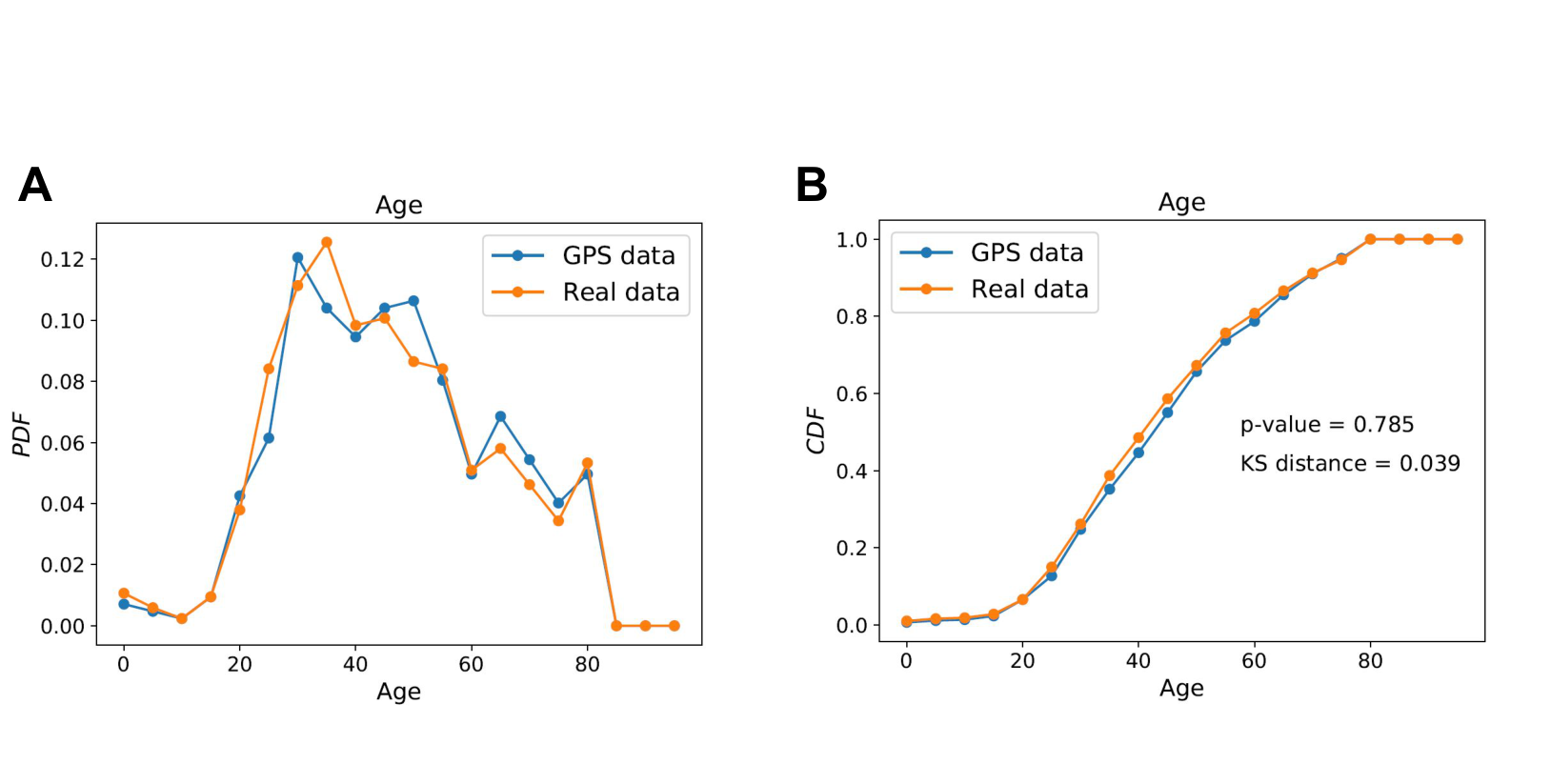

Supplement: S10 Fig — (A) PDF and (B) CDF of age distribution in the GPS geolocalized data compared with the real patient data. We cannot reject the hypothesis that both samples come from the same distribution under KS statistical testing. (TIFF) [file pcbi.1009865.s011.tiff]

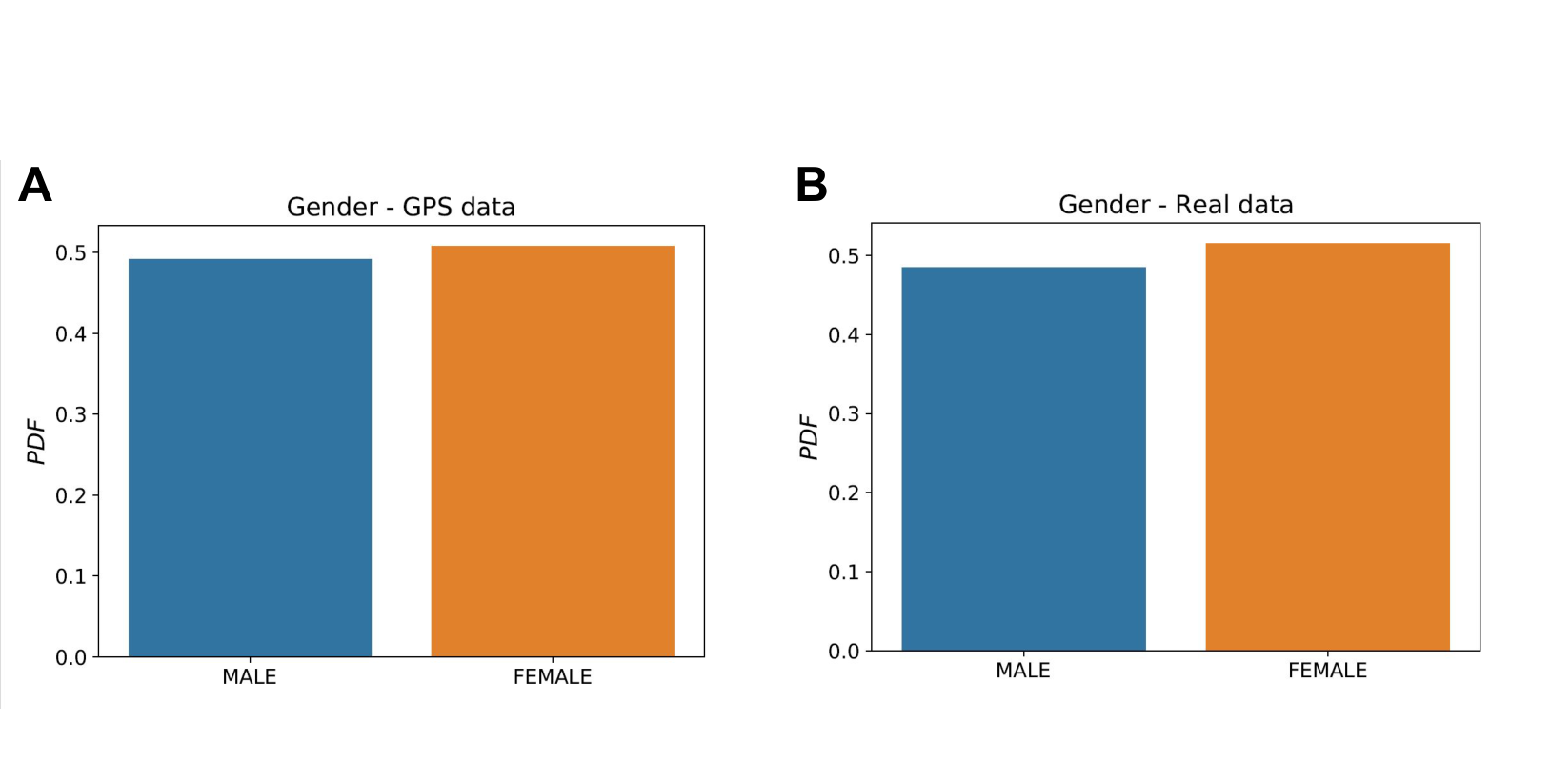

Supplement: S11 Fig — (A) PDF and (B) CDF of gender distribution in the GPS geolocalized data compared with the real patient data suggesting lack of bias. (TIFF) [file pcbi.1009865.s012.tiff]

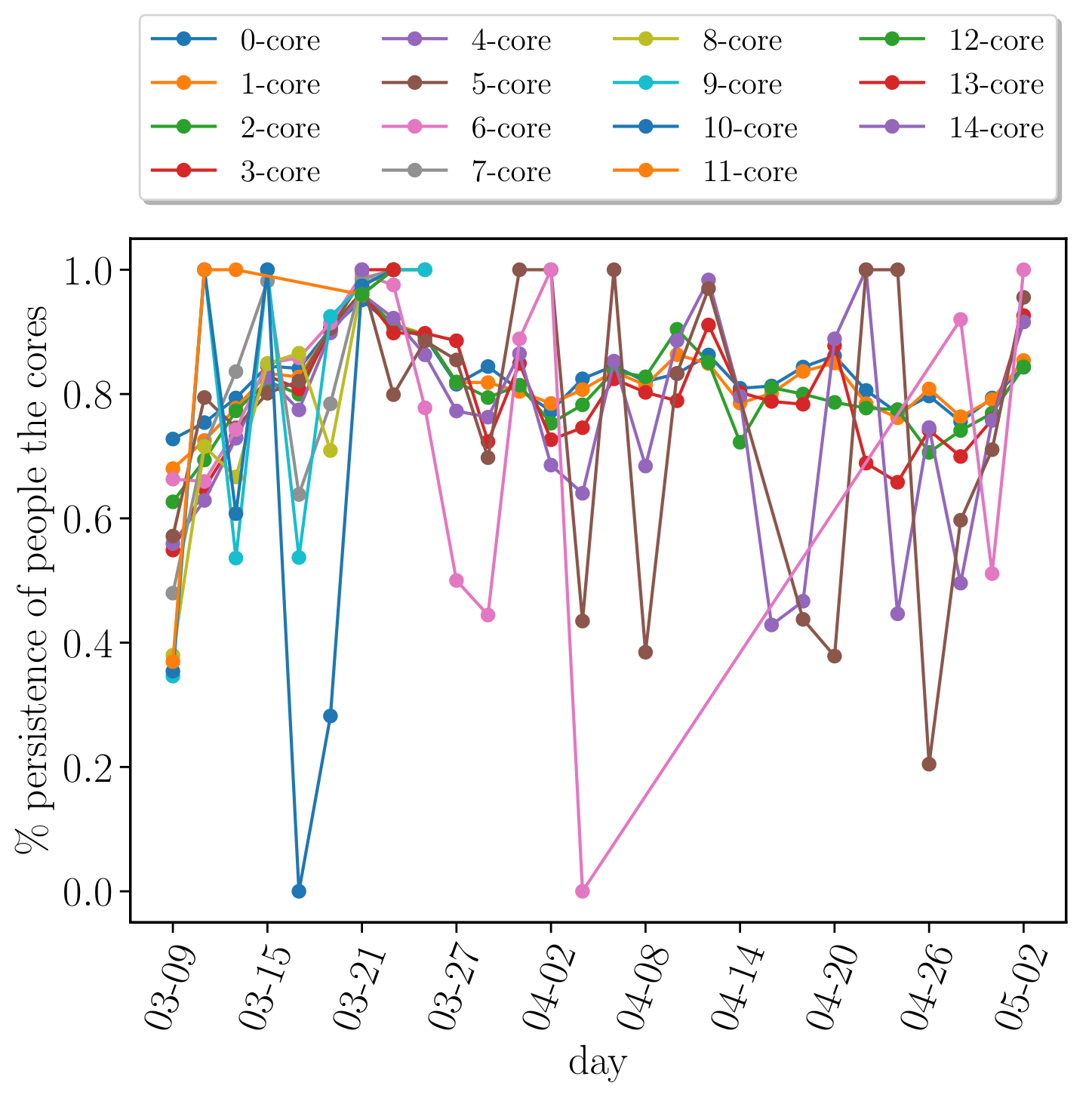

Supplement: S12 Fig — Persistence of people in the k-cores in the temporal networks. We plot the percentage of people in the cores from network to network. The persistance is calculated by the overlap of people in the k-shells from a time of observation to the next (three days later in this particular example). (TIFF) [file pcbi.1009865.s013.tiff]

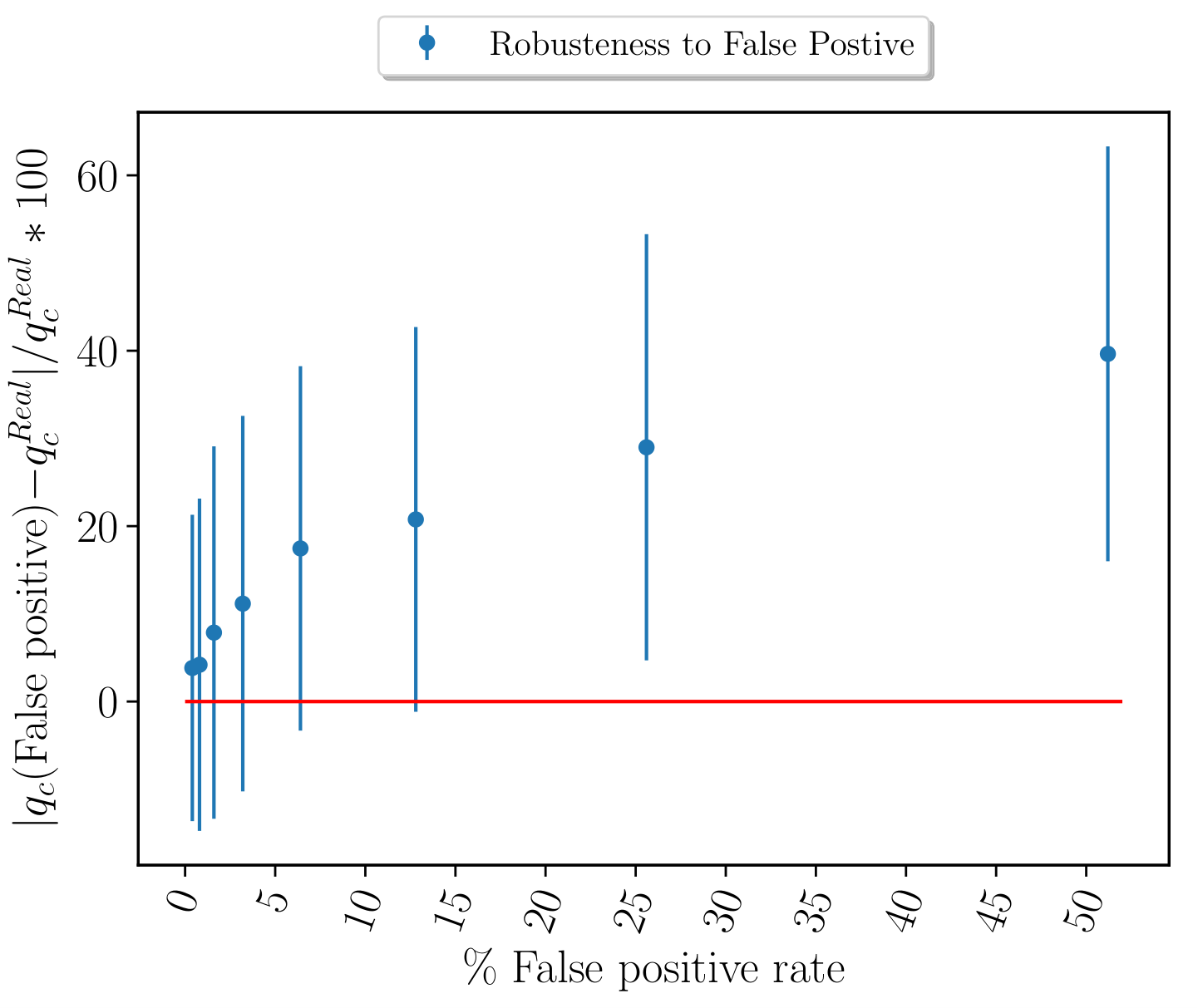

Supplement: S13 Fig — Normalized efficacy of BC centrality as a function of false positives in the report of infected people. A false positive is an individual who reported to have symptoms but was not infected with Covid-19. We plot the relative error in the determination of the minimal number of people to quarantine versus the false positive rate. The measure starts to deviate from linear behaviour beyond the error bars around 20% false positive rate. (TIFF) [file pcbi.1009865.s014.tiff]

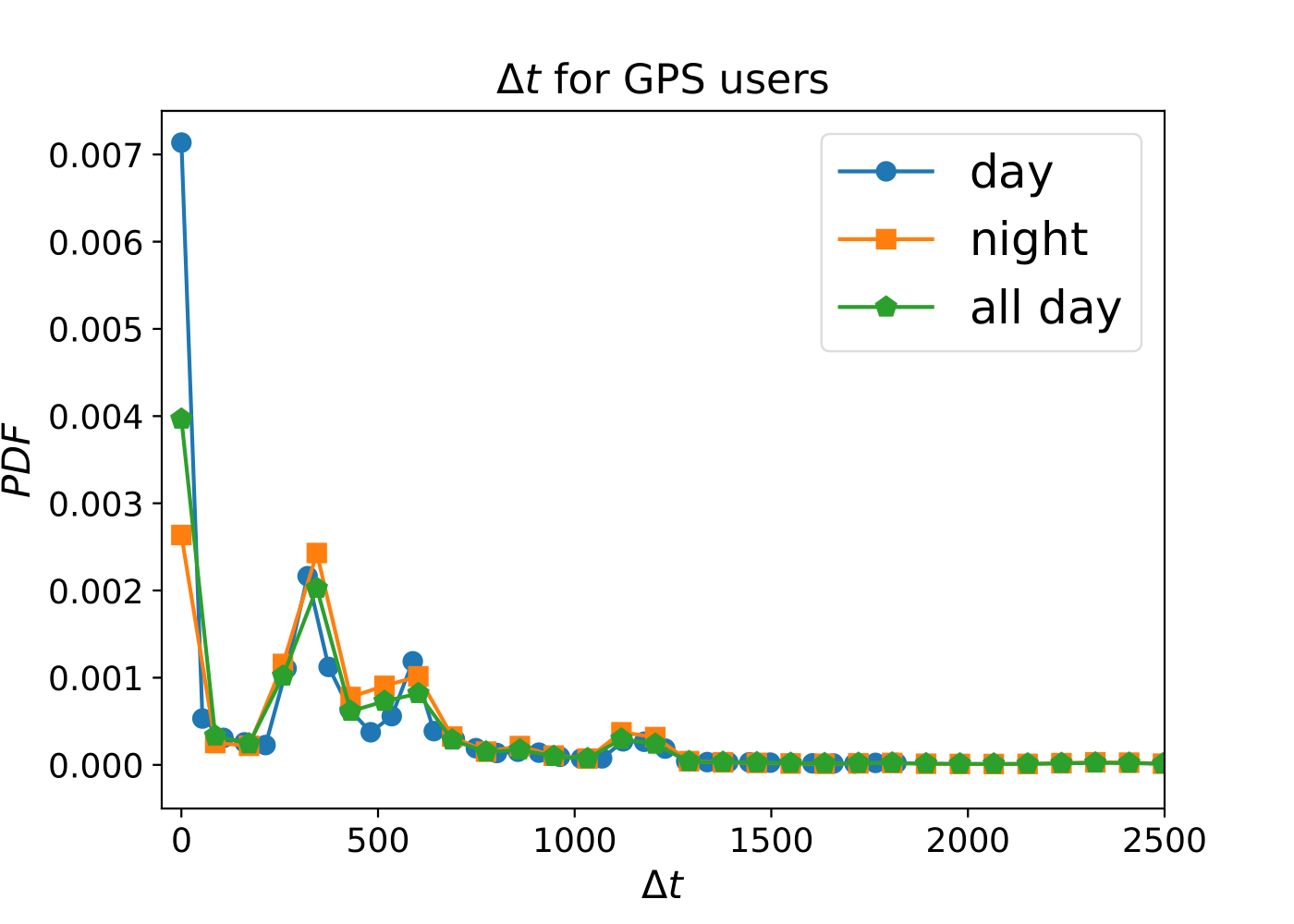

Supplement: S14 Fig — Distribution of the time interval between GPS pings during all day and separated by day and night. (TIFF) [file pcbi.1009865.s015.tiff]

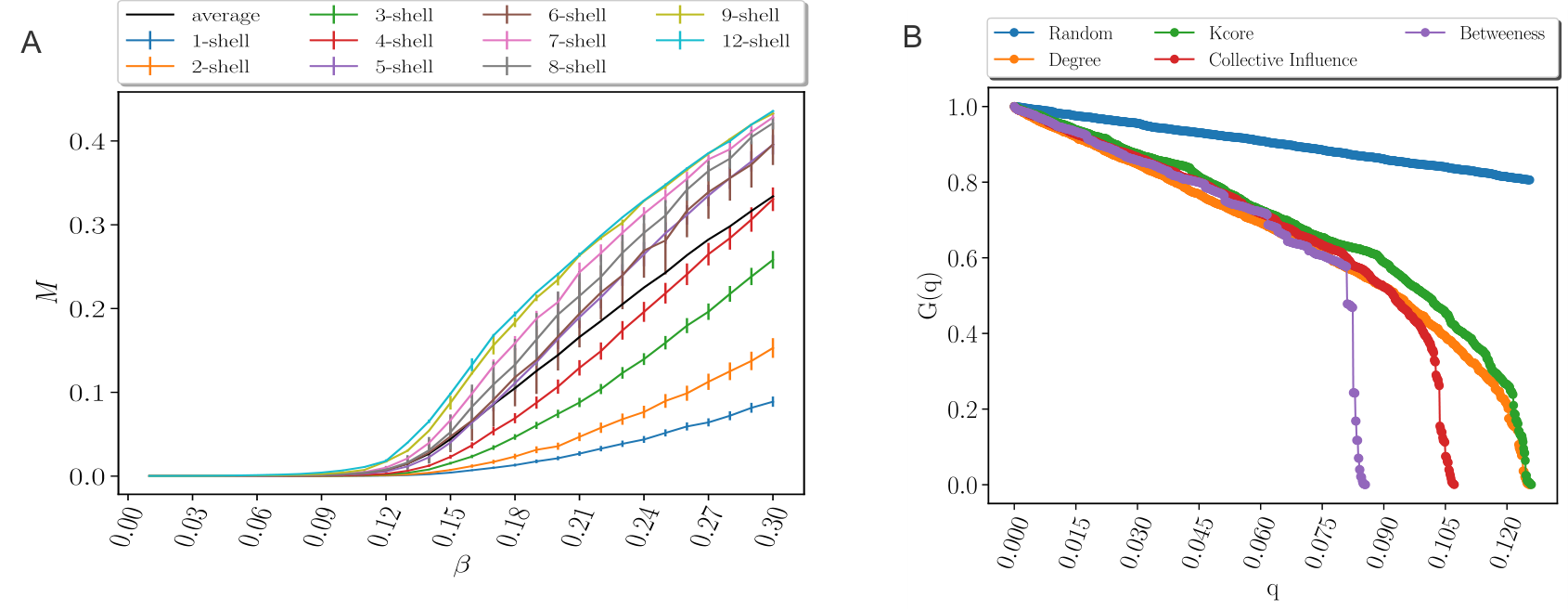

Supplement: S15 Fig — (A) Amount of infected population (M=∑MiN see [33]) when the spreading starts in a given node in a k-shell as a function of the probability of infection β for a SIR model on the same network on March 19 in Fig 3A in pre-quarantine Ceará. The black is the average value over all the starting nodes in the network. The average divides the shell contribution to the spreading of the virus in two groups above and below the average. The 0.5-core composed of the 6-core (kcoremax=12 in this network) which contains nodes from the 6-shell to the 12-shell, has maximal spreading. The 0.5-shell which is composed by the remaining shell from 1-shell to 5-shell has minimal spreading, below the average. (B) Optimal percolation analysis performed over the network in Fig 3A before the quarantine on March 19 in Ceará with different attack strategies and their effect on the size of the largest connected component G(q) versus the removal node fraction, q. Depending on the strategy nodes are removed: randomly (blue), by the highest value of betweenness centrality (green) [38, 39], degree (orange), collective influence (red) [20], and by the highest k-shell followed by high degree inside the k-shell [33]. After each removal we re-compute all the metrics. The best strategy among those studied is removing the nodes directly by the highest value of betweenness centrality. (TIFF) [file pcbi.1009865.s016.tiff]

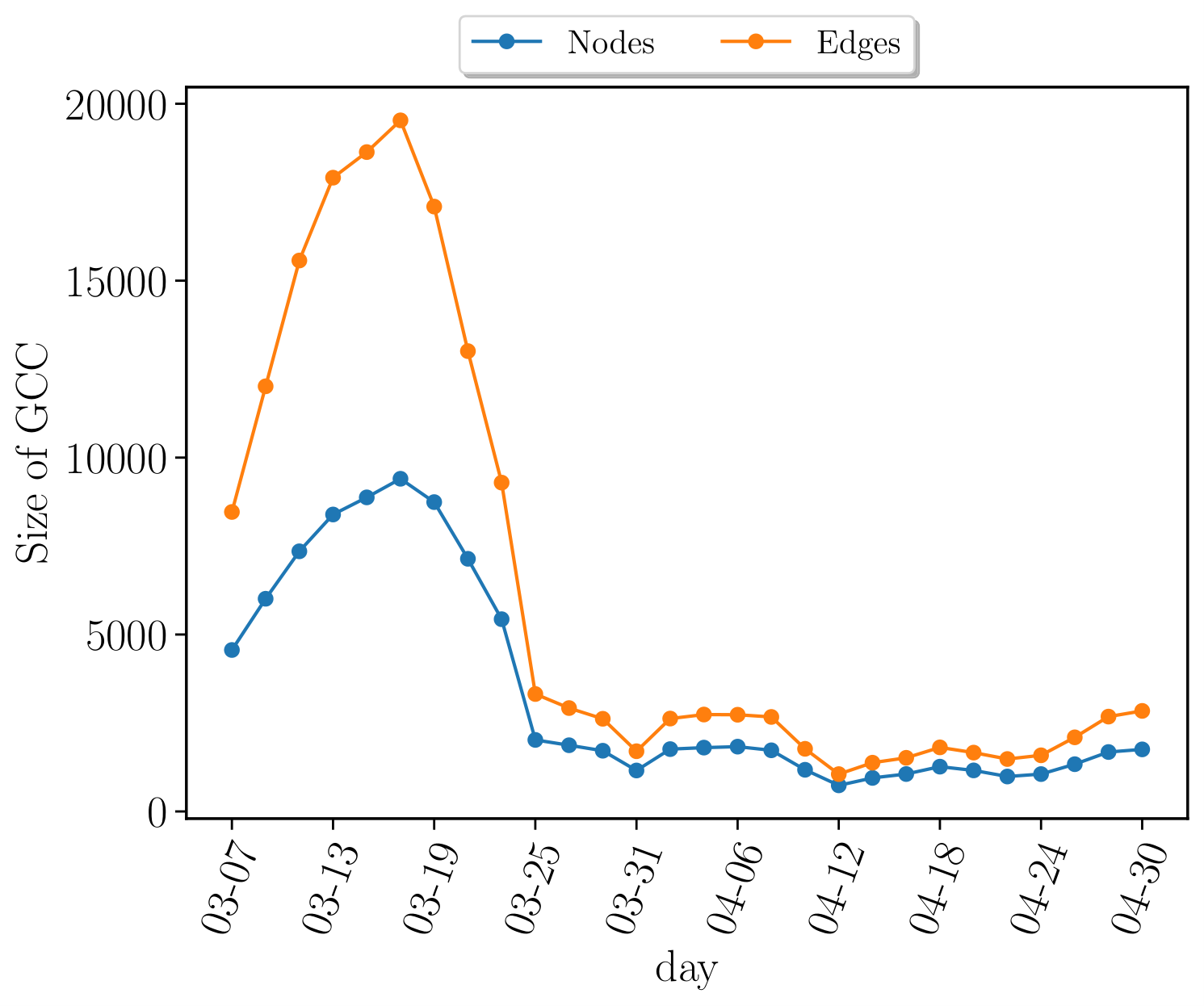

Supplement: S16 Fig — The number of nodes (blue) and edges (oranges) in the GCC versus time. The initial increase in the number of nodes is artificial due to the fact that we perform contact tracing 14 days back for each patient and our data collection started in March 1. Thus the networks in the first two weeks have relatively lower contacts than the rest. (TIFF) [file pcbi.1009865.s017.tiff]

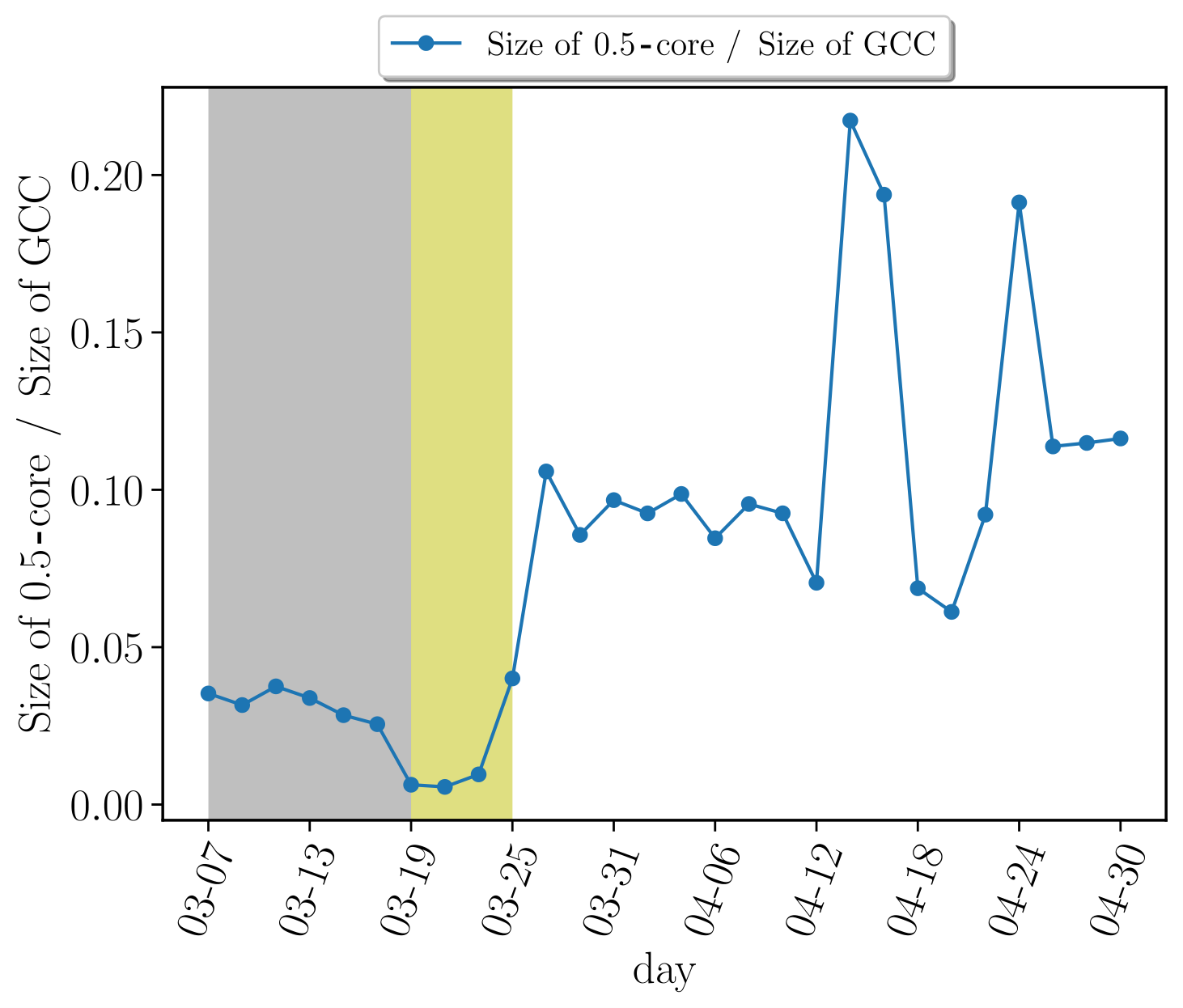

Supplement: S17 Fig — Evolution of maximum 0.5-core size versus time normalized by the size of the GCC. The proportion of these maximum k-cores keeps increasing after the quarantine. (TIFF) [file pcbi.1009865.s018.tiff]
