# Supplementary material for: Digital contact tracing and network theory to stop the spread of COVID-19 using big-data on human mobility geolocalization
Source: PLoS Comput Biol. 2022 Apr 11;18(4):e1009865. doi: 10.1371/journal.pcbi.1009865 (PMC9053778; doi:10.1371/journal.pcbi.1009865)
Supplement: Attachment — Submitted filename: report.pdf [file pcbi.1009865.s019.pdf]

Report

Title: Superspreading k-cores at the center of COVID-19 pandemic persistence

Journal: PLOS Computational Biology

Summary

The manuscript conducts a contact tracing network analysis matching GPS and confirmed cases data applied to the case of COVID-19. They propose an effective way to break the network of transmission based on data from the state of Ceará, in Brazil. This GPS data allow monitoring the mobility of users and build a contact network identifying temporal changes before and after the lockdown and the persistence of k-cores, linked to super-spreading events. The main finding is that it is possible to break the transmission tree quarantining those with high betweenness centrality, linking the maximum k-cores with the rest of the population. The work is original and of high importance, with rigorous network and statistical analyses, very well documented in the Supplementary Material.

The authors state that the research followed ethical guidelines to treat personal data, not allowing to identify anyone, and has the approval of the Epidemiological Surveillance Department of Ceará.

Some points

- I had some questions about the sampling bias and the fact that only a few confirmed cases were linked to the GPS data. I suggest empathizing that these analyses were also performed and cite the Supplementary Material more often in the main text.
- line 18: typo -> “teh state”
- line 20: “both states”? Only Ceará is mentioned before. As I could see in the SM, there is a “Puebla” state that was not used. By the way, in the caption of Table S1 “Puebla” is also mentioned.
- line 40: I suggest changing $\sim$ by $\approx$ if the authors agree.
- line 49: what is the difference between timestamp n and time t?
- lines 151-158: in Ref. DOI:10.1103/PhysRevE.98.012310 the authors show that the maximum k-core can be the driver of disease transmission in contact networks. I was wondering if it has relation with the case presented here.
- How is the degree distribution $P(k)$ of the network? Does it keep the shape over time, taking snapshots aggregating the network over different time windows before and after the lockdown?
- page 25 of SM: typo? -> “These GSP-based apps”
- Notation: The notation about k-cores and k-shells can be improved. Sometimes they appear as 1-shell (line 128) or 1 k-shell (Fig. 4a), while 0.5-kcore is also used. I would suggest to use 1-shell and 0.5-core, instead, to match the k-shell and k-core pattern, respectively.
