# Supplementary material for: Digital contact tracing and network theory to stop the spread of COVID-19 using big-data on human mobility geolocalization
Source: PLoS Comput Biol. 2022 Apr 11;18(4):e1009865. doi: 10.1371/journal.pcbi.1009865 (PMC9053778; doi:10.1371/journal.pcbi.1009865)
Supplement: Attachment — Submitted filename: Response_To_Reviewers.pdf [file pcbi.1009865.s020.pdf]

City College of New York

Hernán A. Makse
Levich Institute and Department of Physics
140th St. and Convent Ave.
New York, NY 10031

Telephone: (212) 650-6847 Telefax: (212) 650-6835
E-mail: hmakse@ccny.cuny.edu

November 12, 2021

RE: *PCOMPBIOL-D-21-00586*
Dr. Thomas Leitner
Deputy Editor
PLOS Computational Biology

Dear Dr. Leitner:

Thank you very much for obtaining the referee reports of our manuscript. After carefully reviewing your editorial as well as the referee's observations, we would like to resubmit the paper for your consideration.

Below we append a point-by-point response to Referee #1 and #3 and the corrections to the revised manuscript.

We look forward to hearing from you.
Sincerely yours,

Hernán Makse

Detailed response to Reviewer #1

This paper implements a comprehensive contact tracing network analysis to find an optimized quarantine protocol to dismantle the chain of transmission of coronavirus with minimal disruptions to society. The authors track billions of anonymized GPS human mobility data points to monitor the evolution of the contact network of disease transmission before and after mass quarantines. The results reveal here are timely and interesting. However, there are some issues that need to be clarified.

We thank the reviewer for the encouraging words.

Two data sets are chosen to be studied in the paper. One is the Grandata united nations development program partnership to combat covid and the other is an anonymized list of confirmed covid patients obtained by the health department authorities from the two countries. There are some mobility data available covering a wide range of regions out there. The authors may want to comment on the choice of the datasets and limit their conclusion about the findings.

We thank the reviewer for pointing out these points. We have added the following paragraph in the article's introduction, Page 2, describing previous work on mobility studies and our choice of the dataset. We comment on the limits of our approach below in the paragraph below.

"Mobile phones or similar devices provide digital sources of information on human mobility and therefore offer a promising way to automate outbreak location detection. Mobile datasets generally consist of an ID associated with each user, a timestamp of the user location, and a location provided as latitude/longitude, which places the user in space. In [1] the authors propose a method to identify outbreak locations of point-source outbreaks from geo-located GPS movement data of affected individuals as recorded from mobile phones. In [2] the authors investigate whether the observed discrepancies between mobile phone datasets affect the results of epidemic simulations. Ferretti *et al.* [3] showed that a contact tracing App can achieve epidemic control if used by enough people without resorting to mass quarantines. Other works combined cross-sectional survey and GPS data. For example, in [4] the authors define a contact tracing strategy that is likely to identify a sufficient proportion of infected individuals such that subsequent spread could be prevented. The solutions proposed often rely on using GPS data alone or combining GPS with self-reported infections (through a mobile app or questionnaire). Our study uses two complementary datasets. The first includes data from 'Grandata-United Nations Development Programme partnership to combat COVID-19 with data' [5]. It is composed of anonymized global positioning system (GPS) data from a compilation of hundreds of mobile applications (apps) across Latin America that allow to track the trajectories of people (users). The data identify each mobile phone device with a unique encrypted mobile ID and specifies its latitude and longitude location through time, which is encoded by a geohash with 12 digits precision. Typically, this dataset generates $\approx$ 450 million data points of GPS location per day across Latin America (see Methods). Our analysis is focused on the state of Ceará, Brazil, where we track the geolocation of over a quarter million unique users generating over half a billion GPS datapoints during the three months period of our study. The second dataset is an anonymized list of confirmed COVID-19 patients obtained from the Health Department authorities from the City of Fortaleza, Ceará, Brazil. The dataset contains the geohash of the residential address, the SARS-CoV-2 test detection date, and the first day of symptoms for each patient infected with COVID-19 in the city of Fortaleza over the studied period, which starts with patient zero arriving in the city and being detected on March 8, 2020. This dataset is used with the consent of the local health authorities in Fortaleza, Ceará and constrained the possibility of retrieving the chain of transmission of the virus to the state of Ceará. We cross-match the location of the residential address of each patient with the GPS geolocation from the mobile phone dataset, thus obtaining the encrypted mobile ID of the patients (see Methods). We then trace the geolocalized trajectories of COVID-19 patients during a period -14/+7 days from the onset of symptoms to look for contacts of the infected person. These contacts define the chain of transmission of the disease which is obtained using the model described below."

We limit the conclusions about our findings with the following paragraph added to the conclusions section, Page 9 (notice that this paragraph answers also the question below):

"It is worth stressing that the optimal strategy to break the transmission of the virus depends on the particular spreading dynamics of the disease, patterns of mobility, and strength of the quarantine applied to each region. As we show in Fig. 4B, every centrality measure can, with a certain degree of disruption, dismantle the chain of transmission of the virus. As we can see from the same figure, the betweenness centrality provides the minimal number of nodes that need to be isolated to dismantle the chain of transmission as compared with the studied centralities. The reason why BC performs better than the other centralities can be found in the particular structure of the contact network left after the quarantine. As we show in Fig. 4C a k-core structure appears due to the strict lockdown, during which only essential workers were allowed to go out. The lockdown essentially removes the majority of the links leaving only those inside the k-cores plus their weak links. These k-cores, which represent the virus reservoir, are generally located in hospitals, warehouses, and some particular condominiums since they are composed mainly of the essential workers who are allowed to circulate during the quarantine. The k-cores are connected by a few links, which work as bridges for the virus transmission. This particular network structure explains why a BC-based ranking is able to break the transmission chain with fewer removals than other centralities, since BC can identify better those bridges that connect the k-cores.

Thus, in the particular case of Fortaleza, we found that betweenness centrality provides the best ranking among the studied centralities to break the transmission chain. However, in another pandemic or even the same pandemic under a different quarantine protocol, the particular network structure that we found in Fortaleza may not appear. Therefore we do not expect that BC will always be the best method to break the transmission chain, and each particular case should be analyzed independently.

However, the strategy proposed here to use contact tracing and network theory is valid for any pandemic. This includes building and monitoring the GCC of transmission as a function of time by combing GPS data with patient-list data and then testing different centralities with the objective of finding the best strategy to break the GCC. Each pandemic and quarantine may lead to a different network structure with its concomitant optimal centrality. The proposed protocol is then to investigate all centralities as done in this study and find the strategy that would break the chain of transmission in the most optimal way."

The infectiousness period of an infected person starts two days before and lasts up to five days after the onset of symptoms. However, this is not directly related to the problem and data studied in this paper. The decision to adding two days to the limits should be commented on.

The idea to add extra days comes about because there is a distribution of the different periods characterizing the disease. Thus, by adding two extra days, we are trying to conservatively capture the range of periods observed in the field, see for instance the work of He *et al.* [6]. We clarify this point with the following paragraph in Page 4:

"In this paper we added two extra days to be conservative in capturing the contacts since the number of days comes from statistical estimations of the different periods characterizing the epidemiological profile of the disease, see Ref. [6]."

Both cc and k-core have been studied in this paper. However, it is recently reported that a generalized k-core is more suitable for the study of spreading behavior. Therefore, this aspect should be discussed and compared thoroughly.

We thank the referee for pointing out this work. We implemented a Generalized k-core percolation strategy [7] and substitute the old Fig. 4B in the main paper with Fig. 1 below. We added the following paragraph on Page 8 of the revised manuscript:

"A generalized k-core strategy, which consists in sequentially removing the nodes in the k-leaf (where $k = k_{max}$), has been recently reported to be more suitable to study spreading behavior [7]. Figure 1 shows that, in this case, it performs similarly like k-core. The reason for this can be found in the tree structure of the network and its low average degree."

Another concern is regarding the optimal quarantine. The inconsistencies between different centralities should be explained better. Do you expect the same qualitative results for other similar infectious diseases or covid related data in other capabilities? In other words, how general are the obtain results? The resilience of the method should not be overlooked.

We thank the reviewer for the excellent advice. As we show in Fig. 4B, every centrality measure can, with a certain degree of disruption, dismantle the chain of transmission of the virus. As we can see from the same figure, the betweenness centrality (BC) provides the minimal number of nodes that need to be isolated to dismantle the chain of transmission as compared with other centralities. The reason why BC performs better than the other centralities can be found in the structure of the contact network left after the quarantine. As we show in Fig. 4C a k-core structure appears due to the strict lockdown during which only essential workers were allowed to go out. These k-cores, which represent a virus reservoir, are generally located in hospitals, warehouses, and some particular condominiums and are connected by a few links, which work as bridges for the virus transmission. This particular network structure explains why a BC ranking is able to break the transmission chain with fewer removals than other centralities. Indeed, BC can identify better those bridges that connect the k-cores. We added the following paragraph in the conclusion section of the revised paper, Page 9 (the paragraph is also explained above in response to the first question of the referee):

"It is worth stressing that the optimal strategy to break the transmission of the virus depends on the particular spreading dynamics of the disease, patterns of mobility, and strength of the quarantine applied to each region. As we show in Fig. 4B, every centrality measure can, with a certain degree of disruption, dismantle the chain of transmission of the virus. As we can see from the same figure, the betweenness centrality provides the minimal number of nodes that needs to be isolated to dismantle the chain of transmission as compared with the studied centralities. The reason why BC performs better than the other centralities can be found in the particular structure of the contact network left after the quarantine. As we show in Fig. 4C a k-core structure appears due to the strict lockdown, during which, only essential workers were allowed to go out. The lockdown essentially removes the majority of the links leaving only those inside the k-cores plus their weak links. These k-cores, which represent the virus reservoir, are generally located in hospitals, warehouses, and some particular condominiums since they are composed mainly of the essential workers who are allowed to circulate during the quarantine. The k-cores are connected by a few links, which work as bridges for the virus transmission. This particular network structure explains why a BC-based ranking is able to break the transmission chain with fewer removals than other centralities, since BC can identify better those bridges that connect the k-cores.

Thus, in the particular case of Fortaleza, we found that betweenness centrality provides the best ranking among the studied centralities to break the transmission chain. However, in another pandemic or even the same pandemic under a different quarantine protocol, the particular network structure that we found in Fortaleza may not appear. Therefore we do not expect that BC will always be the best method to break the transmission chain, and each particular case should be analyzed independently.

However, the strategy proposed here to use contact tracing and network theory is valid for any pandemic. This includes building and monitoring the GCC of transmission as a function of time by combing GPS data with patient-list data and then testing different centralities with the objective of finding the best strategy to break the GCC. Each pandemic and quarantine may lead to a different network structure with its concomitant optimal centrality. The proposed protocol is then to investigate all centralities as done in this study and find the particular strategy that would break the chain of transmission in the most optimal way."

Detailed response to Reviewer #2

The presented manuscript proposed an interesting contention strategy for the spreading phenomena in contact networks obtained from real GPS human mobility data. The authors create a contact tracing network that presumes to have the full contagion network. Then, they conclude k-core structures persist in the transmission network even when an extreme measure such as a lockdown is applied maintaining the spreading activity. This suggests that an optimized isolation measure can be found to avoid contagion. After trying different centrality measures the authors found that the betweenness centrality is the best breaking the transmission network. I found this preprint very interesting, well-written, and easy to follow. Furthermore, it contains enough novel results to be published in PLOS Computational Biology.

We are glad the reviewer appreciated our work and we thank her/him for the encouraging words.

Detailed response to Reviewer #3

The manuscript conducts a contact tracing network analysis matching GPS and confirmed cases data applied to the case of COVID-19. They propose an effective way to break the network of transmission based on data from the state of Ceará, in Brazil. This GPS data allow monitoring the mobility of users and build a contact network identifying temporal changes before and after the lockdown and the persistence of k-cores, linked to super-spreading events. The main finding is that it is possible to break the transmission tree quarantining those with high betweenness centrality, linking the maximum k-cores with the rest of the population. The work is original and of high importance, with rigorous network and statistical analyses, very well documented in the Supplementary Material. The authors state that the research followed ethical guidelines to treat personal data, not allowing to identify anyone, and has the approval of the Epidemiological Surveillance Department of Ceará. I had some questions about the sampling bias and the fact that only a few confirmed cases were linked to the GPS data. I suggest empathizing that these analyses were also performed and cite the Supplementary Material more often in the main text.

We thank the reviewer for the suggestion. We added in the revised manuscript more references to the Supplementary Material:

Page 4: (see Supplementary Material, section S7.20)

Page 4: (see Supplementary Material, section S2)

Page 5: (see Supplementary Material, section S3)

Page 5: (see Supplementary Material, section1 S7.1)

Page 5: (see Supplementary Material, section S4)

Page 7: (see Supplementary Material, section S5)

Page 7: (see Fig. S15A in the Supplementary material, section S15)

line 18: typo – > "teh state"

We apologize for the typo. We have corrected it in the revised version of the manuscript.

line 20: "both states"? Only Ceará is mentioned before. As I could see in the SM, there is a "Puebla" state that was not used. By the way, in the caption of Table S1 "Puebla" is also mentioned.

We apologize for the confusion. In the paper we have focused on the state of Ceará, only. We removed the references to Puebla in the revised manuscript. We added the following paragraph in Page 2 to clarify:

"Our analysis is focused in the state of Ceará, Brazil where we track the geolocation of over a quarter million unique users generating over half a billion of GPS datapoints during the three months period of our study."

line 40: I suggest changing $\sim$ by $\approx$ if the authors agree

We thank the reviewer for the suggestion. We changed the symbol in the revised version of the manuscript.

line 49: what is the difference between timestamp n and time t?

The timestamp n enumerates each GPS datapoint. n has an "enumerative" meaning while t_n refers to the actual time attached with that point. We clarify this in Page 4 with the following sentence:

"The timestamp n enumerates each GPS datapoint, while t_n refers to the actual time attached with that point."

lines 151-158: in Ref. DOI:10.1103/PhysRevE.98.012310 the authors show that the maximum k-core can be the driver of disease transmission in contact networks. I was wondering if it has a relation with the case presented here.

Indeed, Ref. DOI:10.1103/PhysRevE.98.012310 the authors showed that the maximum k-core can be a driver of transmission [8]. We have cited this reference in Page 5 (notice that the number of the reference in the main manuscript is 26):

"The highly connected modules found in Fig. 3B and 3C are k-core structures [23–26] of higher complexity than the GCC (which is a 1-core), that are known to sustain an outbreak even when the GCC has been disintegrated [9, 26]."

How is the degree distribution $P(k)$ of the network? Does it keep the shape over time, taking snapshots aggregating the network over different time windows before and after the lockdown?

The reviewer raised an interesting point. We added a section titled "Degree distribution of the contact networks" in the Supplementary Material, Page 12:

"We analyzed the degree distribution of the contact networks before and after the quarantine. We found a relatively broad distribution as it is shown in Fig. 2, both before and after the quarantine. In addition, we find that after the quarantine the distribution is less broad than before the quarantine, displaying a smaller maximum degree. This finding is consistent with the fact that the lockdown restricts the mobility with a subsequent decrease in the maximum degree of the nodes."

page 25 of SM: typo? $->$ "These GSP-based apps"

We apologize for the typo. We have corrected it in the revised version of the manuscript.

Notation: The notation about k-cores and k-shells can be improved. Sometimes they appear as 1-shell (line 128) or 1 k-shell (Fig. 4A), while 0.5-kcore is also used. I would suggest to use 1-shell and 0.5-core, instead, to match the k-shell and k-core pattern, respectively

We apologize for the misleading notation. In the revised manuscript we followed the suggestion of the reviewer.

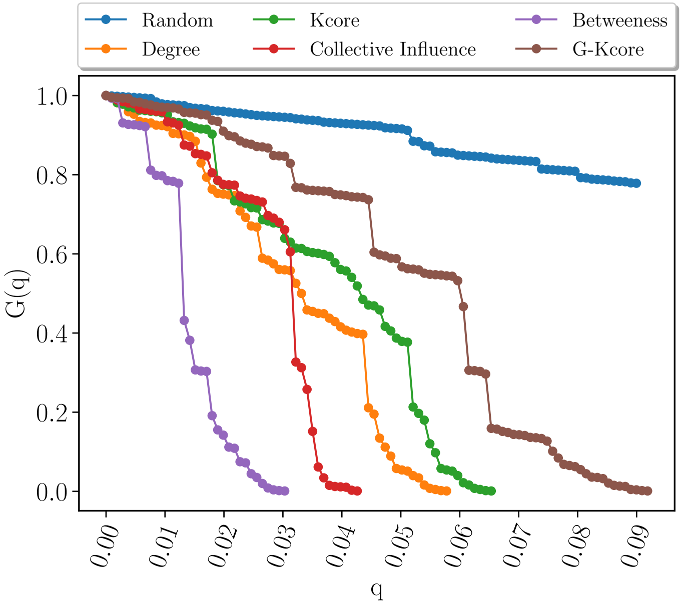

FIG. 1: Optimal percolation analysis performed over the network in Fig. 3C during the lockdown in following different attack strategies and their effect on the size of the largest connected component G(q) versus the removal node fraction, q. Nodes are removed (in order of increasing efficiency): randomly (blue); by the highest k-shell followed by high degree inside the k-shell [22]; by highest degree(orange); by collective influence (red) [13]; by the highest generalized k-core (brown) [7]; and by the highest value of betweenness centrality (green) [27, 28]. After each removal we re-compute all metrics. The most optimal strategy among those studied is removing the nodes by the highest value of betweenness centrality.

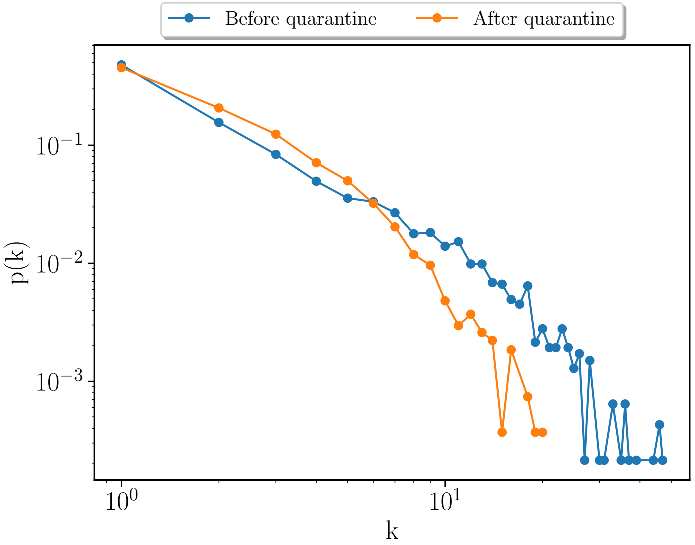

FIG. 2: Degree distribution of the contact network before (blue) and after (orange) the quarantine.

[1] Frank Schlosser and Dirk Brockmann. Finding disease outbreak locations from human mobility data. *EPJ Data Science*, 10(1):1–17, 2021.

[2] Michele Tizzoni, Paolo Bajardi, Adeline Decuyper, Guillaume Kon Kam King, Christian M Schneider, Vincent Blondel, Zbigniew Smoreda, Marta C González, and Vittoria Colizza. On the use of human mobility proxies for modeling epidemics. *PLoS computational biology*, 10(7):e1003716, 2014.

[3] Luca Ferretti, Chris Wymant, Michelle Kendall, Lele Zhao, Anel Nurtay, Lucie Abeler-Dörner, Michael Parker, David Bonsall, and Christophe Fraser. Quantifying sars-cov-2 transmission suggests epidemic control with digital contact tracing. *Science*, 368(6491), 2020.

[4] Matt J Keeling, T Deirdre Hollingsworth, and Jonathan M Read. Efficacy of contact tracing for the containment of the 2019 novel coronavirus (covid-19). *J Epidemiol Community Health*, 74(10):861–866, 2020.

[5] Grandata-united nations development programme partnership to combat covid-19 with data. `https://covid.grandata.com`. Accessed 9 Nov 2021.

[6] Xi He, Eric HY Lau, Peng Wu, Xilong Deng, Jian Wang, Xinxin Hao, Yiu Chung Lau, Jessica Y Wong, Yujuan Guan, Xinghua Tan, et al. Temporal dynamics in viral shedding and transmissibility of covid-19. *Nature medicine*, 26(5):672–675, 2020.

[7] N Azimi-Tafreshi, S Osat, and SN Dorogovtsev. Generalization of core percolation on complex networks. *Physical Review E*, 99(2):022312, 2019.

[8] Maksim Kitsak, Lazaros K Gallos, Shlomo Havlin, Fredrik Liljeros, Lev Muchnik, H Eugene Stanley, and Hernán A Makse. Identification of influential spreaders in complex networks. *Nature physics*, 6(11):888–893, 2010.
